# Chromosome-level genome assembly and population genomics of Mongolian racerunner (*Eremias argus*) provide insights into high-altitude adaptation in lizards

**DOI:** 10.1186/s12915-023-01535-z

**Published:** 2023-02-20

**Authors:** Weiming Li, Juan Du, Lingyun Yang, Qiqi Liang, Mengyuan Yang, Xuming Zhou, Weiguo Du

**Affiliations:** 1 Key Laboratory of Animal Ecology and Conservation Biology, Institute of Zoology, Chinese Academy of Sciences, Beijing, 100101 China; 2 University of Chinese Academic of Sciences, Beijing, China; 3 Novogene Bioinformatics Institute, Beijing, 100083 China; 4 Center for Excellence in Animal Evolution and Genetics, Chinese Academy of Sciences, Kunming, 650223 China

**Keywords:** Lizard, Whole-genome sequencing, Population genomics, High-altitude adaptation

## Abstract

**Background:**

Although the extreme environmental adaptation of organisms is a hot topic in evolutionary biology, genetic adaptation to high-altitude environment remains poorly characterized in ectothermic animals. Squamates are among the most diverse terrestrial vertebrates, with tremendous ecological plasticity and karyotype diversity, and are a unique model system to investigate the genetic footprints of adaptation.

**Results:**

We report the first chromosome-level assembly of the Mongolian racerunner (*Eremias argus*) and our comparative genomics analyses found that multiple chromosome fissions/fusions events are unique to lizards. We further sequenced the genomes of 61 Mongolian racerunner individuals that were collected from altitudes ranging from ~ 80 to ~ 2600 m above sea level (m.a.s.l.). Population genomic analyses revealed many novel genomic regions under strong selective sweeps in populations endemic to high altitudes. Genes embedded in those genomic regions are mainly associated with energy metabolism and DNA damage repair pathways. Moreover, we identified and validated two substitutions of *PHF14* that may enhance the lizards’ tolerance to hypoxia at high altitudes.

**Conclusions:**

Our study reveals the molecular mechanism of high-altitude adaptation in ectothermic animal using lizard as a research subject and provides a high-quality lizard genomic resource for future research.

**Supplementary Information:**

The online version contains supplementary material available at 10.1186/s12915-023-01535-z.

## Background

An important area in evolutionary biology is understanding the way in which animals have adapted to extreme environment. For example, high altitudes bring multiple challenges, including low oxygen, high ultraviolet radiation, low pressure, and cold temperatures for organisms. However, species that have adapted to such harsh environments usually exhibit numerous physiological capabilities, such as increased heart rates, augmentation of pulmonary ventilation, and increased affinity of hemoglobin for oxygen [1–3]. Previous studies have focused on mammals and birds [4–14] and several genes have been identified by genomic studies, such as *EPAS1*, *MTHFR*, and *EGLN1* in humans [5, 6, 12], *EPAS1* in dogs and horses [9], *TRPC1*, *KCNMA1*, and *SHEEPV1R18* in pigs [8], *SOCS2* in sheep [11], and *SLC35F1* in Tibetan chickens [10]. These genes are the targets of natural selection in high-altitude environments, however, we have a limited understanding of the way in which ectothermic animals such as amphibians and reptiles respond to high altitudes [15, 16].

Squamates, comprising lizards and snakes, have diverse morphological characters and lifestyles, and therefore, have been widely used as research models in evolution and adaptation studies [17–21]. While physiological ecologists have explained and described the biophysical and biochemical patterns of high-altitude adaptation in squamates, the genetic mechanisms underlying the high-altitude adaptive process are still not widely known [22, 23]. Among squamate species, Mongolian racerunner (*Eremias argus*) is the lizard that is extensively distributed in Russia, Korea, Mongolia, and China over a wide range of elevations. This species is currently the only known lizard species that is distributed from the Eastern Plain to the Qinghai-Tibet Plateau in China. Such species could serve as a model for the investigation of genetic adaptation to high altitude in lizards. Here, we first report a chromosome-level genome assembly of the Mongolian racerunner generated using a combination of the latest sequencing and scaffold technologies, including single-molecule real-time, linked-read, second-generation sequencing, and Hi-C scaffolding. We also sequenced genomes of 61 Mongolian racerunner individuals collected from altitudes ranging from ~ 80 to ~ 2600 m above sea level (m.a.s.l.). Using these genome resources, we sought to discover the genome variations that respond to high-altitude adaptation in Mongolian racerunners. Our study provides preliminary evidence for extreme environmental adaptation and expands our knowledge of the genome characteristics in lizards.

## Results

### Genome assembly and chromosomes evolution

**Fig. 1:**
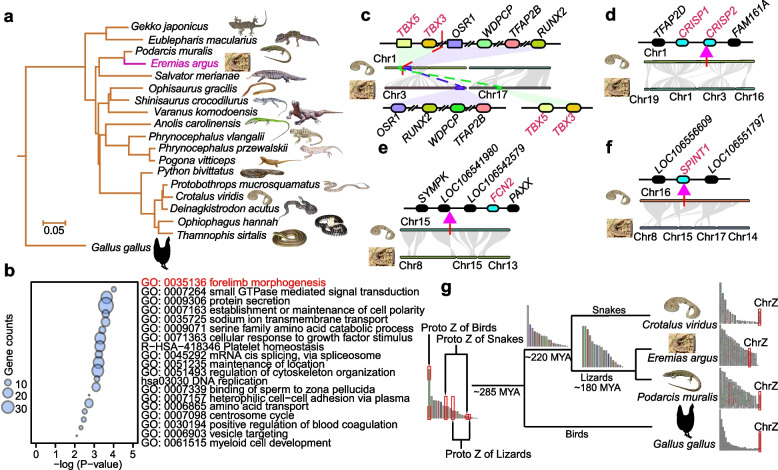
Phylogenetic analysis and chromosome synteny of squamate genomes. **a** Phylogenomic tree constructed using common single-copy orthologous genes from 12 lizards and six snakes, with the chicken as the outgroup. The Mongolian racerunner is highlighted in magenta. All animal pictures are from http://reptile-database.reptarium.cz [26], modified in Adobe Illustrator. **b** Functional enrichment result of Mongolian racerunner genes located at chromosome breakpoint regions caused by chromosome fissions/fusions. **c** Six genes (*RUNX2*, *TFAP2B*, *OSR1*, *WDPCP*, *TBX5*, and *TBX3*) which have a role in limb development were detected in the chromosome breakpoint regions. **d**–**f** Four genes (*CRISP1*, *CRISP2*, *FCN2*, and *SPINT1*) which have a role in rattlesnake venom formation were detected in the chromosome breakpoint regions. **g** The reconstruction of ancestral chromosomes in squamates and chicken genomes. The Chr Z of three species originated from five different autosomes

We generated a 1.63-Gb genome assembly of the Mongolian racerunner using a sample from the desert in Hohhot, Inner Mongolia Autonomous Region of China. We used a combination of the four latest sequencing technologies: single-molecule real-time sequencing (PacBio Sequel), paired sequencing (Illumina HiSeq), linked-read sequencing (10X Genomics), and Hi-C (Phase Genomics, Inc.). This new genome assembly produced 19 chromosomes with contig N50 of 3.07 Mb and scaffold N50 of 96.73 Mb (Fig. 1a; Additional file 1: Figure S1–4; Additional file 2: Table S1–2). Karyotypic analysis demonstrated that the Mongolian racerunner possesses a ZW/ZZ sex determination system, and the two sex chromosomes differ in size and shape [24, 25]. To identify the potential Z chromosome in our chromosome-scale assembly, we compared the sequence coverage between male and female individuals and determined chromosome 15 as the Z chromosome (Additional file 1: Figure S5).

To verify the completeness of our assembly, we checked the Core Eukaryotic Genes (CEGs) using CEGMA [27] and Benchmarking Universal Single-Copy Orthologs (BUSCOs) using BUSCO [28]. This analysis recovered 226 (91.13%) complete CEGs and 2441 (94.39%) the complete single-copy orthologs (Additional file 2: Table S3-4), indicating that the Mongolian racerunner genome has a high coverage of the coding region. We also checked the accuracy of the assembly of this genome using Illumina short reads and obtained a mapping rate of 98.65% and a mapping coverage rate of 99.74% (Additional file 2: Table S5), suggesting that the assembled genome has good consistency. Tandem repeat sequences and interspersed repeat sequences account for approximately 47.37% of the Mongolian racerunner genome assembly, and 46.92% of them are transposable elements (Additional file 1: Figure S6; Additional file 2: Table S6). Long interspersed nuclear elements (LINEs) are the most abundant element, making up approximately 33.18% of the genome assembly. Using a comprehensive annotation strategy, 20,107 protein-coding genes were predicted (Additional file 2: Table S7), with an average length of 28.72 kb, an average CDS length of 1.43 kb, an average of 8.63 exons per gene, and an average intron length of 3.57 kb (Additional file 1: Figure S7). Most of the predicted genes (~ 99.68%) were functionally annotated against known protein databases (Additional file 2: Table S8).

In order to characterize genome synteny between the Mongolian racerunner genome and its relatives, pairwise synteny comparisons were conducted between the Mongolian racerunner, rattlesnake, European common wall lizard, and chicken genomes. Multiple chromosome fissions/fusions events were observed in autosomes and sex chromosomes (Additional file 1: Figure S8-9). For autosomes, genes located at chromosome breakpoint regions (e.g., *RUNX2*, *TFAP2B*, *OSR1*, *WDPCP*, *TBX5*, and *TBX3*) of the Mongolian racerunner genome assembly are enriched in “forelimb morphogenesis,” “embryonic forelimb morphogenesis,” “limb morphogenesis,” and “hindlimb morphogenesis” (Fig. 1b). These limb morphogenesis related genes are located on Chr1 in the snake genome, while their orthologs in the lizard genome are located at different chromosomes: i.e., four genes (*RUNX2*, *TFAP2B*, *OSR1*, and *WDPCP*) are located on Chr3, and two genes (*TBX5* and *TBX3*) were transferred to Chr17 (Fig. 1c). Genes located at chromosome breakpoint regions of the rattlesnake were enriched in “cellular response to organic cyclic compound” (Additional file 1: Figure S10), and four of them (*CRISP1*, *CRISP2*, *FCN2*, and *SPINT1*) encode proteins that are an important part of rattlesnake venom (Fig. 1d–f). Especially, the *FCN2* gene was gained in the snake genome at Chr 15. This gene encodes ficolin which is an essential snake venom component [29, 30]. For sex chromosomes, we found the chicken, snake, and lizard do not share homolog Z chromosome regions (Additional file 1: Figure S11), suggesting that the sex chromosomes of lizards, snakes, and birds might be derived from different autosomal pairs. To explore the origin of Z chromosomes in the Mongolian racerunner and its relatives, we reconstructed 21 ancestral chromosomes of these four species using GRIMM [31]. We found that the chicken ChrZ evolved from one ancestral autosome, lizard ChrZ evolved from two ancestral autosomes, and ChrZ of the snake evolved from another two ancestral autosomes (Fig. 1g**)**. We further checked the location of sex determination-related genes in the lizard genomes, which shows that *DMRT1*, *DMRT2*, and *DMRT3*, the male determining genes in birds [32], are located on the Chr18 of Mongolian racerunner or the Chr17 of European common wall lizard. *SOX3* gene, which is involved in sex determination in mammals [33], is located on ChrZ of the Mongolian racerunner genome and European common wall lizard (Additional file 1: Figure S11). Together, these results suggest that chromosome fissions/fusions occurred frequently and could have contributed to critical evolutionary events in squamates.

### Genome adaptation to high altitude

**Fig. 2:**
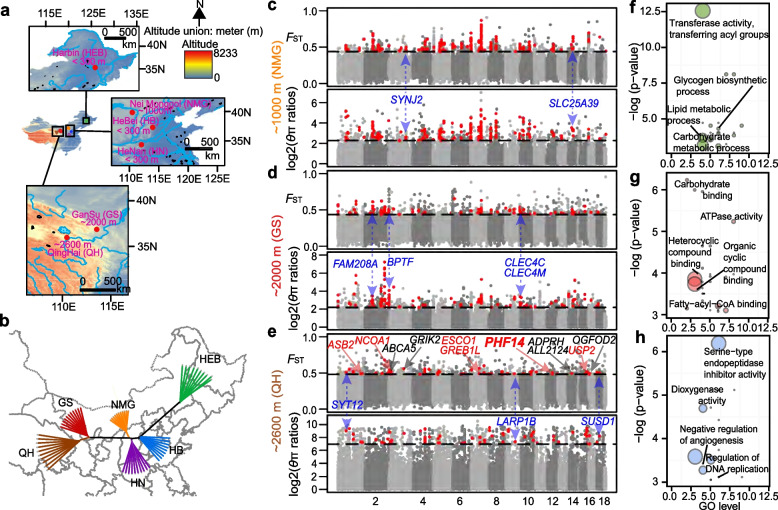
Sampling altitude and selective sweep analysis of different high-altitude adaptations of the Mongolian racerunner. **a** Geographic distribution of the six representative populations. The altitude (m) of the sampling sites in this study is visualized. **b** Neighbor-joining tree constructed from the allele sharing matrix of variants of 61 Mongolian racerunner individuals. The orientations approximately fit the sample geographic locations. The reliability of the neighbor-joining tree was estimated using 100 bootstrap pseudo-replicates. **c**–**e** The panels show the distribution of log_2_(*θ*_π_ ratios) and *F*_ST_ values that were calculated in 40-kb windows and 20-kb steps in mid- and high-altitude populations compared with low-altitude populations. The points in red (corresponding to the top 1% of the log_2_(*θ*_π_ ratios) distribution and the top 1% of the *F*_ST_ value distribution) are genomic regions identified to be under selection in one mid-altitude population (NeiMonggol (NMG, ~ 1000 m)), and two high-altitude populations (GanSu (GS, ~ 2000 m), and Qinghai (QH, ~ 2600 m)). The ~ 1000 m (NMG) population was compared with three low populations (HB, HEB, and HN: altitude under ~ 300 m), the ~ 2000 m (GS) population was compared with three low populations and the NMG population, and the ~ 2600 m (QH) population compared with three low populations, the NMG population, and the GS population. The genes in red functioned by responding to hypoxia or UV, which are important for high-altitude adaptation in lizards. The *PHF14* gene was verified by cell experiments. The explanation of other highlighted genes were shown in Fig. 3 legend. **f–h** The panels show the GO enrichment results (*P* < 0.05 by Fisher exact test) of NMG, GS, and QH positively selected genes. The sizes of the circles represent the number of genes

**Fig. 3:**
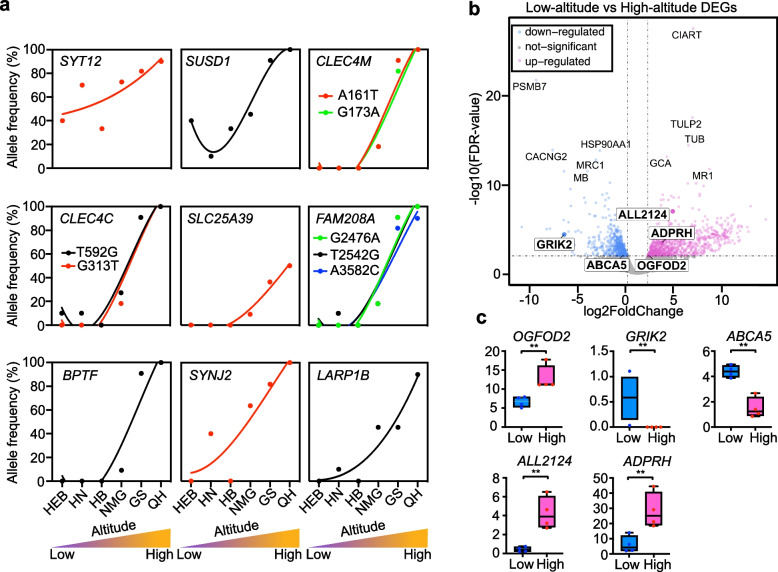
Genotype frequency variation and expression pattern of genes under selective sweeps. **a** The candidate loci that under selective sweep (labeled in blue with boxes shown in Figs. 2c–e) whose genotype frequency increasing with the altitude. The y-axis indicates the allele frequency. The populations on the *x*-axis are ordered by altitude from low (left) to high (right). “Low” and “High” mean low altitude and high-altitude, respectively, including in panel **c**. **b** Volcano plot of differentially expressed genes between the lowest (HB) and highest (QH) altitude populations. Dotted line on *y*-axis represents FDR equal 0.01. Dotted lines on-axis represent FoldChange equal − 2 (left) and 2 (right). Top five down and five up differentially expressed genes are also highlighted. The gene symbols in boxes are candidate loci under selective sweep (labeled in black shown in Fig. 2e) in highest altitude population (QH) who also differentially expressed. **c** The expression pattern of five positively selected genes (*OGFOD2*, *GRIK2*, *ABCA5*, *ALL2124*, and *ADPRH*), which were also highlighted in Fig. 2e and Fig. 3b. *OGFOD2*, *ALL2124*, and *ADPRH* are highly expressed, whereas *GRIK2* and *ABCA5* are lowly expressed (FDR < 0.01). The *y*-axis was FPKM (Fragments Per Kilobase of transcript per Million mapped reads)

To explore the molecular adaptation to mid altitude and high altitude in the Mongolian racerunner, we collected and sequenced six Mongolian racerunner populations from low-altitude to high-altitude areas (Fig. 2a, b; Additional file 2: Table S9-11). We utilized a combination of four parameters (*F*_ST_ and *θ*_π_, XP-CLR, and *D*xy) to identify genomic regions under selective sweeps. We initially used a top 1% *F*_ST_ value and a top 1% *θ*_π_ ratio cutoff to screen the potential selected region in one mid-altitude population (NMG), and two high-altitude populations (GS and QH) compared to low-altitude group (HB, HEB, and HN), respectively (Additional file 1: Figure S12d-f). We identified a total of 179 (*F*_ST_ ≥ 0.44;* θ*_π_ ratio ≥ 2.27), 115 (*F*_ST_ ≥ 0.44;* θ*_π_ ratio ≥ 2.19), and 69 (*F*_ST_ ≥ 0.48;* θ*_π_ ratio ≥ 6.97) genome segments in the NMG, GS, and QH populations relative to the low-altitude group (combined individuals from HB, HEB, and HN populations). These genome segments contained 78, 64, and 55 genes, respectively (Fig. 2c, d). Of these genes, 76.14% (150 out of 197) were also identified under selective sweep using XP-CLR, and 78.68% (155 out of 197) were identified using *D*xy (Additional file 1: Figure S13–15; Additional file 2: Table S12–14). The candidate loci identified by *F*_ST_ and *θ*_π_ ratio among NMG, GS, and QH contrasts showed a low overlapping rate (Additional file 1: Figure S16), revealing a uniqueness at molecular adaptation across populations, which also has been observed in amphibians and reptiles [34]. Among these outlier genes, *TMEM68*, a candidate glycerolipid metabolism gene [35], overlapped in the NMG and GS selective sweep sets, and *ABCA8B*, a member of the ABCA transporter subfamily and associated with sphingolipid metabolism [36], overlapped in GS and QH. Genes under selective sweep in lizards endemic to high altitudes (> 2,000 m) are mainly associated with energy metabolism and DNA damage repair pathways (Fig. 2e–h; Additional file 2: Table S15–20). Interestingly, the allele frequency in 13 nonsynonymous mutations from nine genes increased with an increase in altitude, suggesting that these genes are important for the high-altitude adaptation of lizards (Fig. 3a). For example, *SYT12*, *CLEC4M*, and *CLEC4C* regulate calcium ion binding, may be involved in hypoxia-induced regulation [37–39]. Given that the QH population is under the highest environmental pressure, we further examined the 55 genes in the *F*_ST_ and *θ*_π_ outlier regions between QH and the low-altitude populations. Several genes seem to have helped the Mongolian racerunner adapt to the high altitude based on previous in vivo or in vitro functional experiments: e.g., *ASB2* is related to angiogenesis [40], and *PITPNM2* and *ARHGAP6* are associated with enzymatic activity binding oxygen. Three genes, namely, *CAMKK2*, *CABP5*, and *MICU2*, have important roles in calcium binding and are required for the expression of hypoxia-inducible genes [41]. One gene, *ESCO1*, ensures the correct replication of DNA in hypoxic environments (Additional file 1: Figure S17) [42, 43].

To investigate how changes in gene expression profiles correlate with changes in altitude of Mongolian racerunner, we performed transcriptomic analysis of lung tissues between low and high populations. The genes with high *F*_ST_ and *θ*_π_ values and high XP-CLR score included the upregulated *P2RX4*, *FAM13A*, and *NARF* genes, and the downregulated *NCOA1*, *TMEM168*, and *USP2* genes, which have been suggested to associate with high-altitude adaptation [44–47]. This observation prompted us to explore other genes with expression changes and that may be under selection in Mongolian racerunners. As a result, we identified 493 downregulated and 845 upregulated differentially expressed genes (DEGs) between HB (the lowest altitude population) and QH (the highest altitude population) (Fig. 3b). The DEGs were functionally enriched in small molecule catabolic and organic hydroxy compound metabolic processes (Additional file 1: Figure S18). Of these genes, *OGFOD2*, *GRIK2*, *ABCA5*, *ALL2124*, and *ADPRH* also located in regions with high *F*_ST_ and *θ*_π_, and low Tajima’s *D* (Additional file 1: Figure S19). Although *OGFOD2*, *ALL2124*, and *ADPRH* were significantly upregulated (*P* < 0.01), *GRIK2* and *ABCA5* were significantly downregulated (*P* < 0.01) in the high-altitude population (Fig. 3c). Among these five genes, *GRIK2* encodes a cold-sensing receptor [48], and a reduction in its expression may confer cold resistance in high-altitude Mongolian racerunner populations.

### Functional validation of genetic variants in the *PHF14* gene

**Fig. 4:**
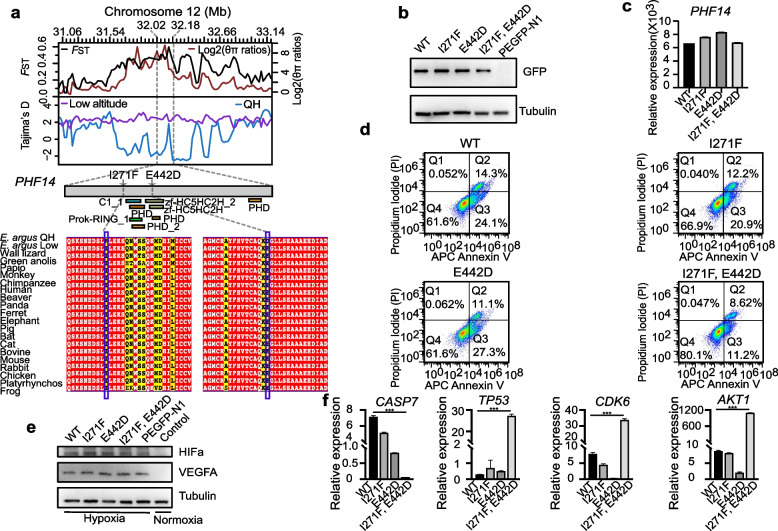
Functional assays of the PHF14 missense SNPs. **a** The log_2_(*θ*_π_ ratios), population differentiations (*F*_ST_), and Tajima’s *D* of selected regions around gene *PHF14* are shown in the top panel. The middle panel shows the structure of the *PHF14* protein. A multispecies alignment of the amino acid sequence of *PHF14* near the mutation point is provided at the bottom. **b** Western-blotting analysis of the HEK293 cell lysates transfected with the GFP-tagged recombinant plasmid of WT, I271F, E442D, and I271F, E442D, or the empty vector. WT, I271F, E442D and I271F, and E442D have plasmids overexpressing the wild-type PHF14 protein, the I271F mutant, the E442D mutant protein and I271F, and the E442D double mutant protein, respectively. PEGFP-N1 represents the empty vector. Anti-GFP and anti-tubulin antibodies were used to measure the protein expression of PHF14 and the internal reference protein tubulin, respectively. **c** The mRNA expression levels of *PHF14* in HEK293 overexpressing cell lines measured by qPCR. **d** Effect of hypoxic stimulus on the apoptosis of HEK293 cells. **e** HIF-1α and VEGFA expression in the WT, I271F, E442D, and I271F, E442D, or PEGFP-N1 overexpressing HEK293 cells under hypoxic conditions was detected by western blotting. Tubulin was used as the internal loading control. **f** Gene expression of the cell apoptosis genes, including *CASP7*, *TP53*, *CDK6*, and *ATK1* in transfected HEK293 cells, measured by qPCR

Gene *PHF14* has a high *F*_ST_ and log2(*θ*_π_), as well as a low Tajima’s *D* value, indicating strong signals of selection (Fig. 4a). *PHF14* is reported to be a novel regulator of mesenchyme growth via platelet-derived growth factor (*PDGF*) receptor-alpha [49, 50] and a hypoxia-sensitive epigenetic regulator in cell cycle progression [51]. *PHF14* is highly expressed in lung cancer, and its high expression is associated with poor survival [51, 52]. Silencing of *PHF14* suppresses cancer proliferation [51–54]. This gene harbored two exonic candidate SNPs in the high-altitude population (QH population). In order to test the potential functional differences of the *PHF14* genotypes (I271F and E442D) in high-altitude lizards compared with the lowland species, we assessed the effects of the I271F and E442D mutations in human embryonic kidney cells (HEK293) under hypoxic conditions. Our functional experiments displayed that the wild-type (WT) *PHF14*, which comes from a low-altitude genotype, and its high-altitude variants exhibited comparable expression, as assessed by western blotting and RT-qPCR (Fig. 4b, c; Additional file 2: Table S21). After culturing the cells for 24 h under hypoxic conditions, the HEK293 cell lines with overexpressed PHF14 variants exhibited lower rates of apoptosis relative to wild-type PHF14, with a double mutant PHF14 (I271F and E442D) showing the lowest rate of apoptosis (*P* < 0.01) (Fig. 4d). Hypoxia-inducible factor 1 (HIF-1α) and vascular endothelial growth factor (VEGFA) are the master regulators of homeostasis under hypoxic conditions [55, 56]. Western blotting showed that the expression of the HIF-1α and VEGFA proteins was significantly increased after a hypoxic stimulus in WT, I271F, E442D, double mutant (I271F and E442D) PHF14, and EGFP overexpressing HEK293 cells (*P* < 0.05) (Fig. 4e). We further investigated whether the PHF14 mutant genotypes could alter the transcriptional response to cell apoptosis using RT-qPCR assays. The mRNA expression levels of three out of the nine downstream genes tested, namely, *TP53*, *CDK6*, and *ATK1*, were significantly increased in the double mutant (I271F and E442D) cells (*P* < 0.01) (Fig. 4f). The mRNA expression levels of *CASP7* were significantly decreased in double mutant (I271F and E442D) PHF14 overexpressing HEK293 cells (*P* < 0.01). No significant changes in mRNA expression levels were observed in single mutants (I271F or E442D). These data suggest that the I271F and E442D may collectively amplify the transcriptional activity mediated by PHF14 in response to apoptosis under hypoxic conditions.

## Discussion

In this study, we investigated the genetic basis of high-altitude adaptation in lizards based on the high-quality reference genome and whole-genome resequencing. We conducted selective sweeping analysis for the populations from different altitudes using a combination method of *F*_ST_, *θ*_π_, XP-CLR, and *D*xy and identified candidate genomic regions with exceptionally high levels of population differentiation on a genome scale. A total of 197 genes were found, showing strong evidence of selection in mid- and high-altitude populations. The *PHF14* gene, displaying reduced genetic diversity and Tajima’s *D* in a high-altitude population, was identified and validated as a target gene of natural selection for hypoxic adaptation in lizards. However, it is worth noting that, due to the limitations of the sample size and the genome scanning methods used in the present study, both additional samples and computational methods are required to evidence high-altitude adaptation in lizards. Furthermore, since mutations of the *PHF14* gene are partially validated, further investigation into genes located in genomic regions under selective sweeps would provide greater insight into mechanisms of adaptation.

## Conclusions

We sequenced and assembled a high-quality genome of the Mongolian racerunner, the lizard distributed from the central and eastern plain to the Qinghai–Tibet Plateau, to understand how lizards adapt to high-altitude environments. Using this genome as reference, we found that chromosome fissions/fusions might be related to limb degeneration, snake venom, and sex chromosome evolution. Subsequent population genomics of Mongolian racerunners from high and low-altitude populations revealed a variety of novel genes associated with local adaptations to high altitude. Taken together, our study provides a high-quality genome assembly of the lizard and furthers our understanding of the genetic basis of high-altitude adaptation using reptiles as a model system.

## Methods

### Samples collecting and preparing

For de novo sequencing and assembly, the muscle tissue of an adult female Mongolian racerunner, which was collected from the desert in Hohhot, Inner Mongolia Autonomous Region of China, was harvested for DNA extraction. For genome annotation, RNA libraries were constructed with six tissues (heart, kidney, gonad, lung, liver, and skin) from the same female adult lizard. A total of additional 61 Mongolian racerunner individuals were collected for whole genome resequencing, containing 10 individuals from Harbin of Heilongjiang province, 9 individuals from Xingtai of Hebei province, 10 individuals from Gongyi, Zhengzhou of Henan province, 11 individuals from Hohhot of Inner Mongolia Autonomous Region, 11 individuals from Jingtai, Baiyin of Gansu province, and 10 individuals from Gonghe, Tibetan Autonomous Prefecture of Hainan of Qinghai province. All the lizards used in this study were euthanized by CO_2_ and tissue samples were first flash frozen in liquid nitrogen and then stored at − 80℃ until use. Total genomic DNA was extracted from muscle tissue by using the stand Phenol–chloroform method.

### Pacific Biosciences (PacBio) library

Genomic DNA was isolated using a Qiagen DNA Genomic kit (Qiagen, Valencia, CA, USA) and the integrity of DNA was checked using agarose gel electrophoresis. DNA purity was checked using the NanoPhotometer® spectrophotometer (IMPLEN, CA, USA). The qualified DNA samples were randomly broken into fragments by g-TUBE (Covaris) and large fragments (≥ 20 kb) of DNA were enriched and purified by magnetic beads. Fragments of DNA were repaired by damage repair and end repair. The stem ring-like adaptors were added at the two ends of DNA fragments, and the fragments that failed to be repaired were removed by exonuclease. Approximately 40-kb SMRTbell libraries were prepared according to the released protocol from PacBio. The single-molecule real-time (SMRT) sequencing was carried out using P6-C4 chemistry on the PacBio Sequel platform at Beijing Novogene Co. Ltd (Beijing, China). An approximate 115.3-fold genome coverage (187.94 Gb) long reads were generated for the assembly.

### Illumina sequencing and quality control

A total of 1.5 µg DNA per sample was used for the DNA sample preparations. Sequencing libraries were generated using Truseq Nano DNA HT Sample preparation Kit (Illumina USA) following the manufacturer’s recommendations and index codes were added to attribute sequences to each sample. Briefly, the DNA sample was fragmented by sonication to a size of 350 bp, then DNA fragments were end polished, A-tailed, and ligated with the full-length adapter for Illumina sequencing with further PCR amplification. At last, PCR products were purified (AMPure XP system) and libraries were analyzed for size distribution by Agilent2100 Bioanalyzer and quantified using real-time PCR.

For Illumina HiSeq sequencing, paired-end libraries were constructed with insert sizes around 350 bp according to Illumina’s protocol. An approximate 65.30-fold genome coverage (106.44 GB) of Illumina paired-end reads was generated for the assembly.

For population genetic analysis, we generated 1399.362 Gb Illumina reads (~ 12.47-average fold per individual). All of the Illumina reads were controlled for quality and sequenced on an Illumina Hiseq sequencer at Beijing Novogene Co. Ltd (Beijing, China). We removed low-quality paired reads as follows: Reads with ≥ 10% unidentified nucleotides (N); > 10 nt aligned to the adaptor, allowing ≤ 10% mismatches; > 50% bases having phred quality < 5; putative PCR duplicates generated in the library construction process.

### 10X Genomics library

The Illumina Read1 sequencing primer, 16 bp barcode, and 6 bp random sequence primer were connected to the GelBeads sequence. The GelBeads passes through two injection ports. The input DNA is mixed with enzymes and dNTP and other reactants after entering the first sample inlet, and the mixture is wrapped by oil drops when passing through the second sample inlet, then it is collected and placed on a special 96-well plate for the barcode library preparation. The random primers were combined with the random position of genomic DNA fragments in the oil drop reaction system for constant temperature PCR amplification. After the library preparation by adding the Illumina P5 and P7 adaptors into the amplification products, PE150 was sequenced on the Illumina platform at Beijing Novogene Co. Ltd (Beijing, China). An approximate 119.10-fold genome coverage (194.14 Gb) 10X Genomics Linked-Reads were generated for the assembly.

### Hi-C library

First, chromatin was digested for 16 h with 400 U HindIII restriction enzyme (NEB) at 37 °C. DNA ends were labeled with biotin and incubated at 37 °C for 45 min, and the enzyme was inactivated with 20% SDS solution. DNA ligation was then performed by the addition of T4 DNA ligase (NEB) and incubation at 16 °C for 4 ~ 6 h. After ligation, proteinase K was added to reverse cross-linking during incubation at 65 °C overnight. DNA fragments were purified and dissolved in 86μL of water. Unligated ends were then removed. Purified DNA was next fragmented to a size of 300–500 bp, and DNA ends were then repaired. DNA fragments labeled by biotin were finally separated on Dynabeads® M-280 Streptavidin (Life Technologies). Hi-C libraries were finally controlled for quality and sequenced on an Illumina Hiseq X Ten sequencer. An approximate 143.25-fold genome coverage (233.50 Gb) Hi-C reads were generated for the assembly.

### Genome assembly

As for the assembly of the genome, the longest PacBio reads were selected as the seed reads, Daligner [57] was used to map all of the PacBio long reads to the seed reads. The pre-assembled reads were obtained by generating a consensus of mapped reads using LASort, LAMerge, and pbdacgon. Unitigs of the pre-assembled reads were used to generate a layout of overlapping reads and the contigs of assembly. The high-quality consensus was obtained after mapping the PacBio long reads to the de novo assembled reference by Quiver [58] (algorithm: arrow). Duplicated assembled haploid contigs were purged using purge_haplotigs [59] with short-insert Illumina reads.

The Linked-reads from the 10X Genomics platform were used to construct a scaffold genome by fragScaff [60]. Illumina reads were used to correct the inaccurate bases in the genome introduced by fragScaff using Pilon [61].

We used Hi-C reads to obtain a chromosome-level genome by Lachesis [62], though fragScaff had improved assemblies and produced a scaffold genome. The frequency of long-range interactions decreases rapidly as the linear distance along a chromosome increases, which helps produce a number of high-quality and contiguous assemblies. The final assembly totaled 1.63 Gb of sequence with a contig N50 of 3.07 Mb and a scaffold N50 of 96.73 Mb.

### Genome annotation

To annotate the repeat sequences of the genome, we initially used LTR_FINDER (version 1.0.7) [63], RepeatModeler (version 2.1), and RepeatScount (version 1.0.5) to find repeats. Next, RepeatMasker (version 4.0.5) [64] was used to search for known and novel transposable elements (TEs) by mapping sequences against the de novo repeat library and Repbase TE library (v20140131). In addition, we used the ProteinMask software (version open-2.1, with parameters: -no LowSimple -*p* value 0.0001) to identify TE-relevant proteins. Overlapping transposable elements belonging to the same type of repeats were integrated together.

We used a homology-based method to annotate the protein-coding gene structure. First, we download genome and annotation information from online database and built a non-redundant protein database of 8 species: *Anolis carolinensis* (ftp://ftp.ensembl.org/pub/release-100/fasta/anolis_carolinensis/dna/Anolis_carolinensis.AnoCar2.0.dna.toplevel.fa.gz), *Pogona vitticeps* (https://ftp.ncbi.nlm.nih.gov/genomes/all/GCF/900/067/755/GCF_900067755.1_pvi1.1/GCF_900067755.1_pvi1.1_genomic.fna.gz), *Shinisaurus crocodilurus* (ftp://parrot.genomics.cn/gigadb/pub/10.5524/100001_101000/100315/Shinisaurus_crocodilurus.fa.gz), *Gekko japonicus* (https://ftp.ncbi.nlm.nih.gov/genomes/all/GCF/001/447/785/GCF_001447785.1_Gekko_japonicus_V1.1/GCF_001447785.1_Gekko_japonicus_V1.1_genomic.fna.gz), *Python molurus bivittatus* (https://ftp.ncbi.nlm.nih.gov/genomes/all/GCF/000/186/305/GCF_000186305.1_Python_molurus_bivittatus-5.0.2/GCF_000186305.1_Python_molurus_bivittatus-5.0.2_genomic.fna.gz), *Protobothrops mucrosquamatus* (https://ftp.ncbi.nlm.nih.gov/genomes/all/GCF/001/527/695/GCF_001527695.2_P.Mucros_1.0/GCF_001527695.2_P.Mucros_1.0_genomic.fna.gz), *Thamnophis sirtalis* (https://ftp.ncbi.nlm.nih.gov/genomes/all/GCF/001/077/635/GCF_001077635.1_Thamnophis_sirtalis-6.0/GCF_001077635.1_Thamnophis_sirtalis-6.0_genomic.fna.gz), and *Deinagkistrodon acutus* (ftp://parrot.genomics.cn/gigadb/pub/10.5524/100001_101000/100196/Deinagkistrodon_acutus.fa.gz). Then the protein sequences were aligned to the genome by using TBlastN [65] with an E-value cutoff by 1E-5. The blast hits were conjoined by solar [66]. For each blast hit, Genewise [67] was used to predict the exact gene structure in the corresponding genomic regions.

For de novo gene prediction, we utilized SNAP (version 2006–07-28) [68], GENSCAN (version 1.0) [69], GlimmerHMM (version 3.0.4) [70], and AUGUSTUS (version 3.1) [71] to analyze the repeat-masked genome. EVidenceModeler software (EVM, version 1.1.1) [72] was used to integrate the genes predicted by the homology and de novo approaches and generate a consensus gene set.

RNA-seq data from 6 issues as mentioned above were mapped to the genome using Tophat (version 2.0.13) [73]. Then cufflinks (version 2.1.1) [74] was used to assemble transcripts to gene models. Transcripts were assembled to expressed sequence tags (ESTs) by Trinity [75] with parameters (-ss 0.5 -jc 0 -minkmercov 2 -minglue 2). We used the transcript information to revise the gene set.

Functional annotation of protein-coding genes was evaluated by BLASTP (*e* value 1E − 05) using two integrated protein sequence databases — SwissProt [76] and NR database. Protein domains were annotated by searching InterPro and Pfam databases, using InterProScan and Hmmer, respectively. Gene Ontology (GO) [77] terms for each gene were obtained from the corresponding InterPro or Pfam entry. The pathways, in which the gene might be involved, were assigned by blast against the KEGG database (release53) [78]. The tRNA genes were identified by tRNAscan-SE [79] software. The rRNA fragments were predicted by aligning to the rRNA sequences database using BlastN at *E*-value of 1E − 10. The miRNA and snRNA genes were predicted by INFERNAL software against the Rfam database [80].

### Phylogenetic tree construction

Gene families were constructed through a hierarchical clustering algorithm and ‘all against all’ BLASTP with a cutoff of 1E − 9 (blast-2.2.26). The alignments with high-scoring segment pairs were conjoined for each gene pair by solar. To identify homologous gene pairs, more than 30% coverage of the aligned regions in both homologous genes was required. Finally, homologous genes were clustered into gene families using the software hcluster_sg.

The phylogenetic tree was reconstructed by using shared single-copy genes. Protein sequences for these single-copy genes were aligned by MUSCLE (version 3.7) [81], then protein sequence alignment was transformed back to CDS alignments. We concatenated the CDS alignments of single-copy genes to a “supermatrix.” Using this supermatrix, we constructed the phylogenetic tree using the ML (maximum likelihood) algorithm as implemented in RAxML software (version 8.0.19) [82].

### Genome synteny

We used MCscan (version v0.8.12) [83] to get synteny blocks among five chromosome-level genomes: Mongolian racerunner, European common wall lizard (*Podarcis muralis*), chicken, green anole lizard, and rattlesnake (*Crotalus viridis*) genomes. We converted the gff files to BED format files, then the CDS sequences were extracted from five genomes and translated into protein sequences as input files. The parameters used for the genome alignment were “–minspan = 10.” The alignments of syntenic chromosomes were visualized by MCscan. The parameters used for visualizing were “–notex.” The chromosome breakpoint regions are defined as the interval between two syntenic blocks from different chromosomes with more than 10 genes in size, which were detected between the Mongolian racerunner and rattlesnake genomes. Ancestral genome reconstruction was performed using GRIMM-Synteny [84] and MGR [31].

### SNP calling and filtering

The high-quality paired-end reads were mapped to the reference genome using BWA (Burrows-Wheeler Aligner) (Version: 0.7.8) [85] with the command “mem -t 4 -k 32 –M.” After alignment, we performed SNP calling on a population scale using a Bayesian approach as implemented in the package SAMtools [86]. We then calculated genotype likelihoods from reads for each individual at each genomic location and the allele frequencies in the sample with a Bayesian approach. The “mpileup” command was used to identify SNPs with the parameters as “-q 1 -C 50 -t SP -t DP -m 2 -F 0.002.” Then, to exclude SNP calling errors caused by incorrect mapping or InDels, only high-quality SNPs (coverage depth ≥ 6 and ≤ 100, RMS mapping quality ≥ 20, maf ≥ 0.1, miss ≤ 0.1) were kept for subsequent analysis. Consequently, 6,303,245 SNPs were left after filtering from 78,513,343 raw SNPs (Additional file 2: Table S11-12). SNP annotation was performed according to the Mongolian racerunner genome using the package ANNOVAR (Version: 2013–05-20) [87].

### Selective sweep and enrichment analysis

We calculated genome-wide distribution of *F*_ST_ values and *θπ* using Vcftools (version 0.1.14) [88] in 40-kb windows with a step size of 20 kb. The *θπ* ratios (*θπ*–*group1*/*θπ–group2*) were calculated for three extreme-control group pairs and log2-transformed. The windows with top 1% *F*_ST_ and -log_2_(θπ ratio), simultaneously were considered as candidate outliers under strong selective sweeps. We also used XP-CLR [89] as a supplement to verify the reliability of the above two selective sweep methods for this project. All outlier regions were assigned to corresponding SNPs and annotated genes. Except above relative divergence methods, we used the absolute divergence (*D*xy) approach to further validate higher divergence regions.

For genes annotated in outlier regions, we used KOBA2.0 [90] software to test the statistical enrichment in KEGG pathways. Gene Ontology (GO) enrichment analysis was implemented by the GOseq R package, in which gene length bias was corrected. GO terms with corrected *P*-value less than 0.05 were considered significant.

The divergent levels (*F*_ST_) of between two subpopulations were simulated by the coalescent simulator ms [91]. We performed the simulations under three isolation with migration (IWM) models. The between-population split time (-ej parameter) and migration rate (-I parameter) were adjusted to match the overall observed *F*_ST_ distributions. The *F*_ST_ values from simulations were calculated by the *evo* program (https://github.com/millanek/evo) with “fst –ms” parameter [92]. The result showed that the top 1% of observed *F*_ST_ (approximately *F*_ST_ ≥ 0.42) are always higher than the corresponding neutral *F*_ST_ values, which is consistent with divergent selection (*F*_ST_) acting on approximately this top 1% of selective sweeping regions.

### Transcriptome sequencing and analysis

For the gene profile of lizard at high altitude, the lungs of four individuals from Qinghai (highest altitude) and four individuals from Hebei (lowest altitude) were harvested locally and preserved in RNA later immediately. A total of 3 μg RNA per sample was used as input material for the RNA sample preparations. Sequencing libraries were generated using NEBNext® UltraTM RNA Library Prep Kit for Illumina® (NEB, USA) following the manufacturer’s recommendations and index codes were added to attribute sequences to each sample. We generated 40.13 Gb RNA-seq reads of six different organ tissues for genome annotation and 55.02 Gb RNA-seq reads of eight lung tissues for transcriptomic analysis of high-altitude adaptation. Index of the reference genome was built using HISAT2-BUILD and the high-quality RNA-seq reads were aligned to the reference genome by the HISAT2 v2.0.4 [93] program with default parameters. HTSeq v0.6.1 [94] was used to count the reads numbers mapped to each gene. And then FPKM (Fragments Per Kilobase of transcript per Million mapped reads) of each gene was calculated based on the length of the gene and reads count numbers. FPKM considers the effect of sequencing depth and gene length for the reads count at the same time, and is currently the most commonly used method for estimating gene expression levels. Differential expression analysis of two conditions (four biological replicates per condition) was performed by using the edgeR [95].

### Cell maintenance and plasmid construct

HEK293 cells were cultivated in DMEM (C11995500BT; Gibco) with 10% FBS (10099141; Gibco) and penicillin (0.2 U/ml)/streptomycin (0.2 lg/ml). The wild-type *PHF14* gene was synthesized in Sangon Company, China. The mutagenesis of the PHF14 sequence (I271F and E442D) was achieved through site-directed mutagenesis and verified by Sanger sequencing. Rapid Recombinant Clone Kit (VI201, TIANGEN) was used to subclone the wildtype PHF14 cDNA, the SNP1 I271F sequence (A to T), or the SNP2 E442D sequence (A to T) into the pEGFP-N1 plasmid for the overexpression analysis.

### Transfection, Western blotting, and qPCR quantification

Transfection of the recombinant plasmids into HEK293 cells was carried out by using Lipofectamine3000 (Invitrogen, America) according to the manufacturer’s instructions. First, 4 μL of Lipofectamine3000 Reagent (Invitrogen, America) and 5 μg of Endofree plasmid were diluted in 125 μL of DMEM (C11995500BT; Gibco). This solution was then mixed with Lipofectamine plasmids Reagent (1:1 ratio) and incubated for 15 min at room temperature, they were then added to 6-well plates (Costar, USA) with 50% cell confluence. These cells were incubated at 37℃ with 5% CO_2_ for 24 h.

Cells were hypoxic stimulus with 1% O_2_ for 24 h after 24 h after transfection and then were washed with cold PBS, harvested, and immediately lysed (RIPA Lysis Buffer, P0013B; Beyotime and PMSF, ST506; Beyotime) for 30 min on ice, cell lysates were centrifuged at 14,000 g for 20 min at 4℃, and supernatants were collected. The total amount of proteins was measured with the BCA method. 10ug of cell lysates supernatants were subjected to western blot with anti-GFP (AE012; Abclonal), anti-HIFα (a16873; ABclonal) anti-VEGFA (ab1316; Abcam), and anti-Tubulin (AC008; Abclonal) antibodies. Antigen–antibody complexes were visualized by enhanced chemiluminescence detection (mageQuantTM LAS 4000).

After the cells were hypoxic stimulus with 1% O_2_ for 24 h, the cells were washed in PBS, harvested, and total RNA was extracted using TransZol Up Plus RNA Kit (ER501-01, TRANS) and RNA was reverse transcribed with GOScriptTM Reverse Transcription System (A5001, Promega) according to the manufacturer’s instructions. Real-time qPCR was performed using SYBR™ Universal PCR Master Mix (439,155; Invitrogen) with first-strand cDNA to evaluate gene expression. Quantitative Real-Time PCR analysis of the TP53, CDK6, AKT1, and CASP7 was carried out using the ABI7500 sequence detection system (Applied Biosystems by Life Technologies, Darmstadt, Germany). The ubiquitous Tubulin gene served as a reference control. PCR primers are shown in Additional file 2: Table S21 and qPCR conditions were as follows: 50 °C for 2 min, initial denaturation at 95 °C for 2 min, 40 cycles of denaturation at 95 °C for 15 s, with combined annealing and extension at 58 °C for 1 min. Three biological replicates were performed per sample.

### Cell apoptosis assays

The HEK293 cells overexpressed WT, I271F, E442D, and I271F, E442D, or the PEGFP-N1 were treated on hypoxic conditions for 24 h. The apoptosis rate was evaluated using the Annexin V-AF647/PI Apoptosis Detection kit (P-CA-203, Procell) according to the instructions from the manufacturer. The cells were seeded into 6-well plates (3 × 10^5^ cells/well). Following hypoxic conditions, the cells were collected, washed with cold PBS, and resuspended in 500 μL binding buffer. Then, 5 μL Annexin V-AF647 and 5 μL PI were added to the buffer and incubated at room temperature for 15 min. Cells were analyzed by flow cytometry (BD FACSCanto) within 1 h.

## Supplementary Information

**Additional file 1:** **Figure S1.** Distribution of 17-mer frequency in Mongolian racerunner genome. **Figure S2.** GC content against the sequencing depths of Mongolian racerunner genome. **Figure S3.** Hi-C interactome plot among Mongolian racerunner chromosomes (Chr1-Chr19). **Figure S4.** Circos plot of the Mongolian racerunner genome assembly showing (from outermost to innermost) Mongolian racerunner chromosomes (Mb), gene density, GC content (%) and TE density (%). **Figure S5.** The female to male depth ratio (log2) for each chromosome. **Figure S6.** Divergence distribution of classified families in Mongolian racerunner genome. **Figure S7.** A comparison of gene parameters among the five lizard genomes. **Figure S8.** Synteny between chicken and Mongolian racerunner genomes (left), and green anole lizard and Mongolian racerunner genomes (right). **Figure S9.** Comparative synteny of chromosomes among European wall common lizard (P. muralis), Mongolian racerunner and rattlesnake (C. viridis) genomes. **Figure S10.** The top 20 functional enrichment results of genes located at rattlesnake genome chromosome breakpoint regions using Metascape. **Figure S11.** Synteny of the Z chromosome among four species. **Figure S12.** The isolation with migration (IWM) models of species formation used for coalescent simulations. **Figure S13.** This figure showed the distribution of XP-CLR values which calculated in 40-kb window and 20-kb step between high and low altitude populations. **Figure S14.** This figure showed the distribution of absolute divergence (Dxy) values which calculated in 40-kb window and 20-kb step between high and low altitude populations. **Figure S15.** The absolute divergence (Dxy) comparison between the top 1% FST. **Figure S16.** Venn plots were shown the global overlaps of the top 1% candidate loci between NMG/GS/QH. **Figure S17.** A concise map of positively selected genes labeled with blue that may relate to hypoxia and UV light response. **Figure S18.** The functional enrichment results of differentially expressed genes (DEGs) using Metascape. **Figure S19.** The FST and θπ, and Tajima’D for the upstream and downstream 1Mb genomic regions of these five genes were calculated in 40kb window. **Figure S20.** The original and uncropped gels for cell experiments.

**Additional file 2: Table S1.** Reads and libraries used in *de novo* Mongolian racerunner genome sequencing. **Table S2.** Assembly statistics of Mongolian racerunner genome. **Table S3.** The CEGMA (Core Eukaryotic Genes Mapping Approach) result of Mongolian racerunner genome. **Table S4.** The BUSCO (Benchmarking Universal Single-Copy Orthologs) result of Mongolian racerunner genome. **Table S5.** The statistics on genome coverage and depth of Mongolian racerunner genome. **Table S6.** The statistics of repeat sequences in Mongolian racerunner genome. **Table S7.** Gene prediction of Mongolian racerunner (*Eremias argus*) genome. **Table S8.** The statistics on functional gene annotation of Mongolian racerunner genome. **Table S9.** Geographical position and sex of sampled Mongolian racerunner individuals. **Table S10.** Mapping statistics of re-sequenced individuals. The genome-wide resequencing data for 61 samples were mapped to the de novo Mongolian racerunner genome. **Table S11.** Summary of SNPs in 61 re-sequenced individuals of Mongolian racerunner. **Table S12.** The top 1% genes under select sweep region in the Inner-Mongolia (NMG) population. **Table S13.** The top 1% genes under select sweep region in the GanSu (GS) population. **Table S14.** The top 1% genes under select sweep region in the Qinghai (QH) population. **Table S15.** Biological Process (BP) GO term enrichment results of selected genes in the NMG populations. **Table S16.** KEGG enrichment results of selected genes in the NMG population. **Table S17.** Biological Process (BP) GO term enrichment results of selected genes in the GS populations. **Table S18.** KEGG enrichment results of selected genes in the GS populations. **Table S19.** Biological Process (BP) GO term enrichment results of selected genes in the QH populations. **Table S20.** KEGG enrichment results of selected genes in the QH populations. **Table S21.** The primers for PCR.

## Data Availability

The raw sequencing data used for genome assembly and whole genome resequencing analysis have been deposited at the public NCBI’s BioProject under the accession number PRJNA659114 (https://www.ncbi.nlm.nih.gov/bioproject/?term=PRJNA659114) [96]. The genome assembly and annotation data have been deposited at the Figshare database (https://doi.org/10.6084/m9.figshare.21098470) [97].
