# Supplementary material for: Chromosome-level genome assembly and population genomics of Mongolian racerunner (Eremias argus) provide insights into high-altitude adaptation in lizards
Source: BMC Biol. 2023 Feb 20;21:40. doi: 10.1186/s12915-023-01535-z (PMC9942394; doi:10.1186/s12915-023-01535-z)
Supplement: Supplementary file 1 — Additional file 1: Figure S1. Distribution of 17-mer frequency in Mongolian racerunner genome. Figure S2. GC content against the sequencing depths of Mongolian racerunner genome. Figure S3. Hi-C interactome plot among Mongolian racerunner chromosomes (Chr1-Chr19). Figure S4. Circos plot of the Mongolian racerunner genome assembly showing (from outermost to innermost) Mongolian racerunner chromosomes (Mb), gene density, GC content (%) and TE density (%). Figure S5. The female to male depth ratio (log2) for each chromosome. Figure S6. Divergence distribution of classified families in Mongolian racerunner genome. Figure S7. A comparison of gene parameters among the five lizard genomes. Figure S8. Synteny between chicken and Mongolian racerunner genomes (left), and green anole lizard and Mongolian racerunner genomes (right). Figure S9. Comparative synteny of chromosomes among European wall common lizard (P. muralis), Mongolian racerunner and rattlesnake (C. viridis) genomes. Figure S10. The top 20 functional enrichment results of genes located at rattlesnake genome chromosome breakpoint regions using Metascape. Figure S11. Synteny of the Z chromosome among four species. Figure S12. The isolation with migration (IWM) models of species formation used for coalescent simulations. Figure S13. This figure showed the distribution of XP-CLR values which calculated in 40-kb window and 20-kb step between high and low altitude populations. Figure S14. This figure showed the distribution of absolute divergence (Dxy) values which calculated in 40-kb window and 20-kb step between high and low altitude populations. Figure S15. The absolute divergence (Dxy) comparison between the top 1% FST. Figure S16. Venn plots were shown the global overlaps of the top 1% candidate loci between NMG/GS/QH. Figure S17. A concise map of positively selected genes labeled with blue that may relate to hypoxia and UV light response. Figure S18. The functional enrichment results of differentially expressed [file 12915_2023_1535_MOESM1_ESM.docx]

**Chromosome-level genome assembly and population genomics of Mongolian racerunner (*Eremias argus*) provide insights into high-altitude adaptation in lizards**

Weiming Li^1,2,5^, Juan Du^1,2,5^, Lingyun Yang^3,5^, Qiqi Liang^3^, Mengyuan Yang^1,2^, Xuming Zhou*^,1^, Weiguo Du*^,1,4^

^1^Key Laboratory of Animal Ecology and Conservation Biology, Institute of Zoology, Chinese Academy of Sciences, Beijing 100101, China.

^2^University of Chinese Academic of Sciences, China.

^3^Novogene Bioinformatics Institute, 100083 Beijing, China.

^4^Center for Excellence in Animal Evolution and Genetics, Chinese Academy of Sciences, Kunming 650223, China.

^5^These authors contributed equally.

*Correspondence and requests for materials should be addressed to W.D. (E-mail: duweiguo@ioz.ac.cn) or to X.Z. (E-mail: zhouxuming@ioz.ac.cn).

**Supplementary Figures**

**
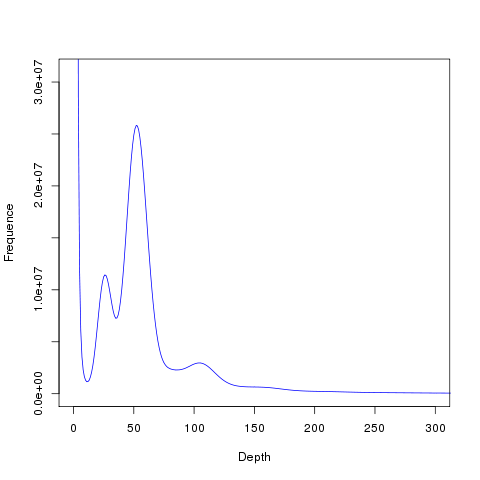
**

**Figure S1.** Distribution of 17-mer frequency in Mongolian racerunner genome. A total of 106.44 Gb of high-quality short-insert reads (350 bp) were used to generate the 17-mer depth curve frequency information.


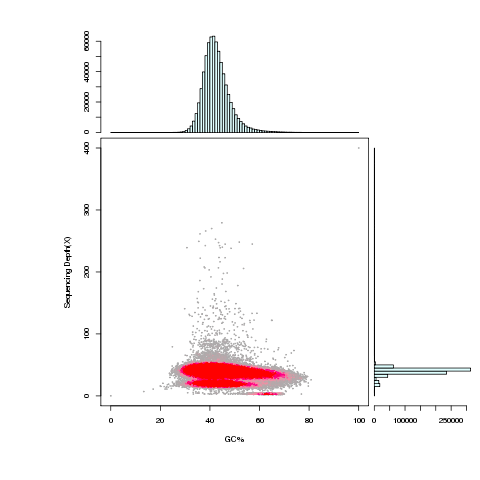


**Figure S2.** GC content against the sequencing depths of Mongolian racerunner genome. The x-axis represents the GC content, and the y-axis represents the average depth. Right panel is the sequencing depth distribution, and above panel is the GC content distribution, while the red part represents the part with a higher density of dots in the scatter plot.

**
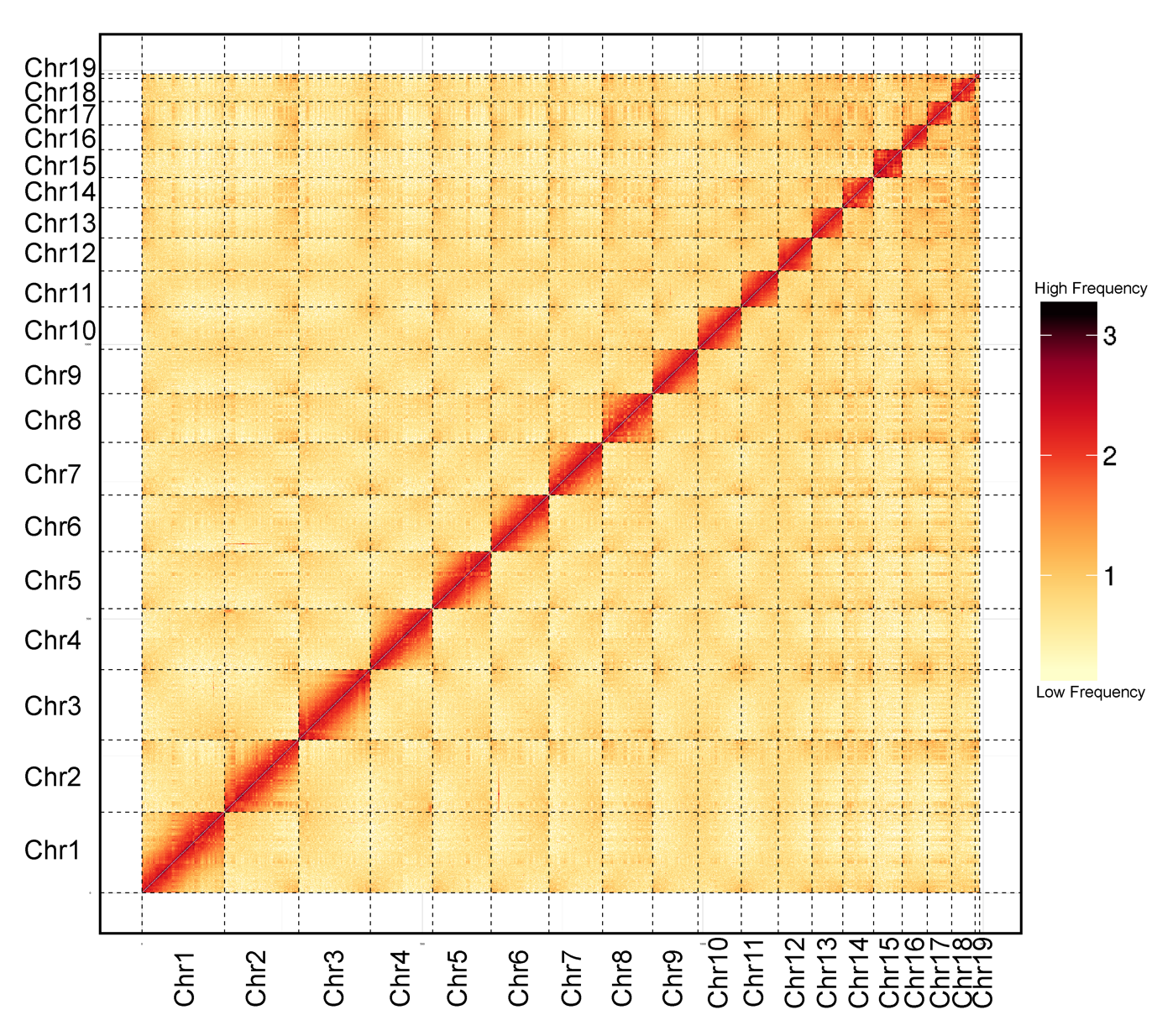
**

**Figure S3.** Hi-C interactome plot among Mongolian racerunner chromosomes (Chr1-Chr19). The interaction frequency was calculated in 1 Mb window. The darker of the color, the stronger of the interaction.


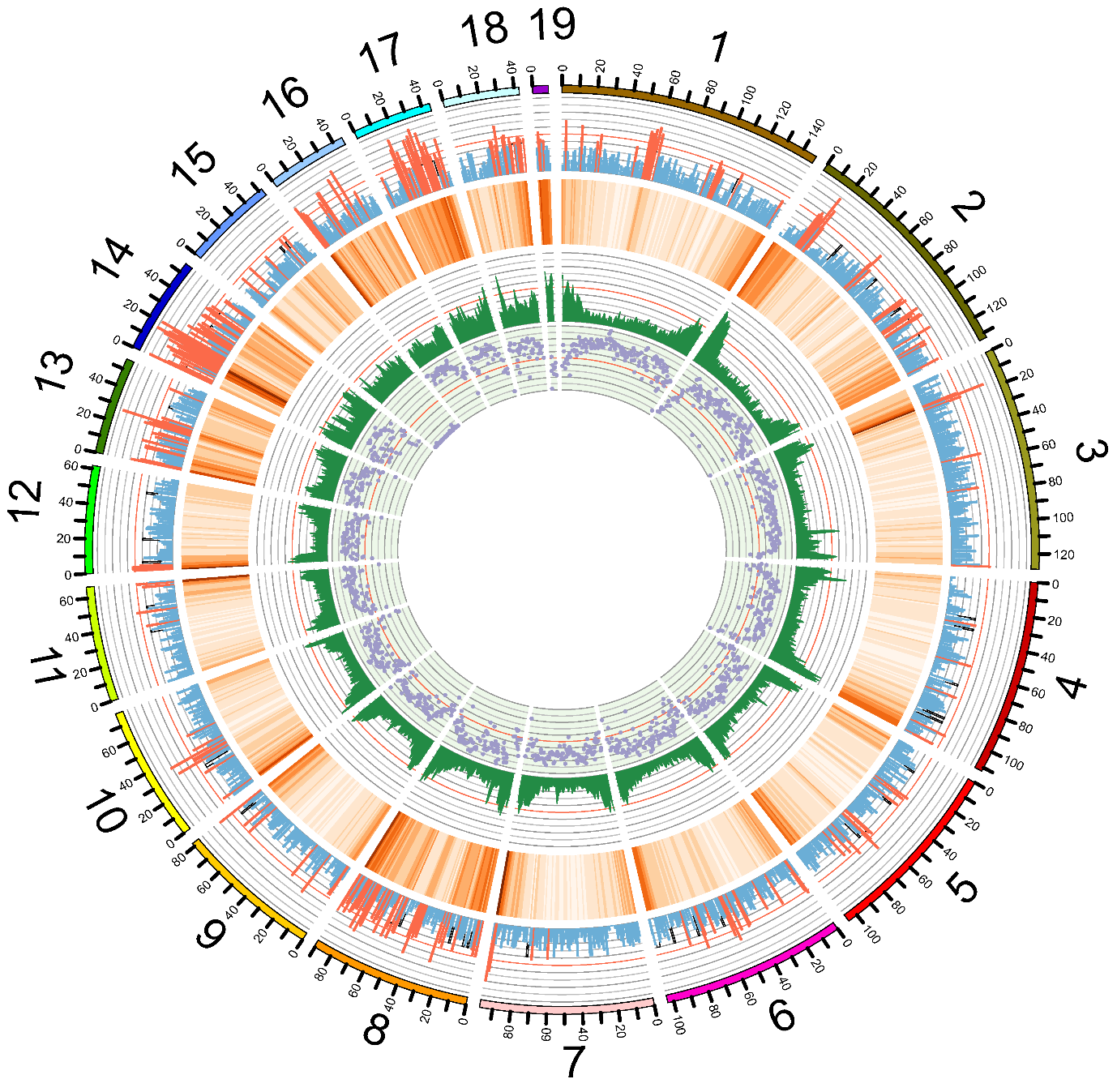


**Figure S4.** Circos plot of the Mongolian racerunner genome assembly showing (from outermost to innermost) Mongolian racerunner chromosomes (Mb), gene density, GC content (%) and TE density (%). This genome assembly consists of 17 autosomes, one sex chromosome (Z, Chromosome 15) and one Microchromosome. Regions of the genome with gene density of more than 20 per 1Mb window are shown in red and less than 20 per 1Mb window shown in blue. The darker color GC content regions represent higher GC. All the data were calculated in 1 Mb window.

**
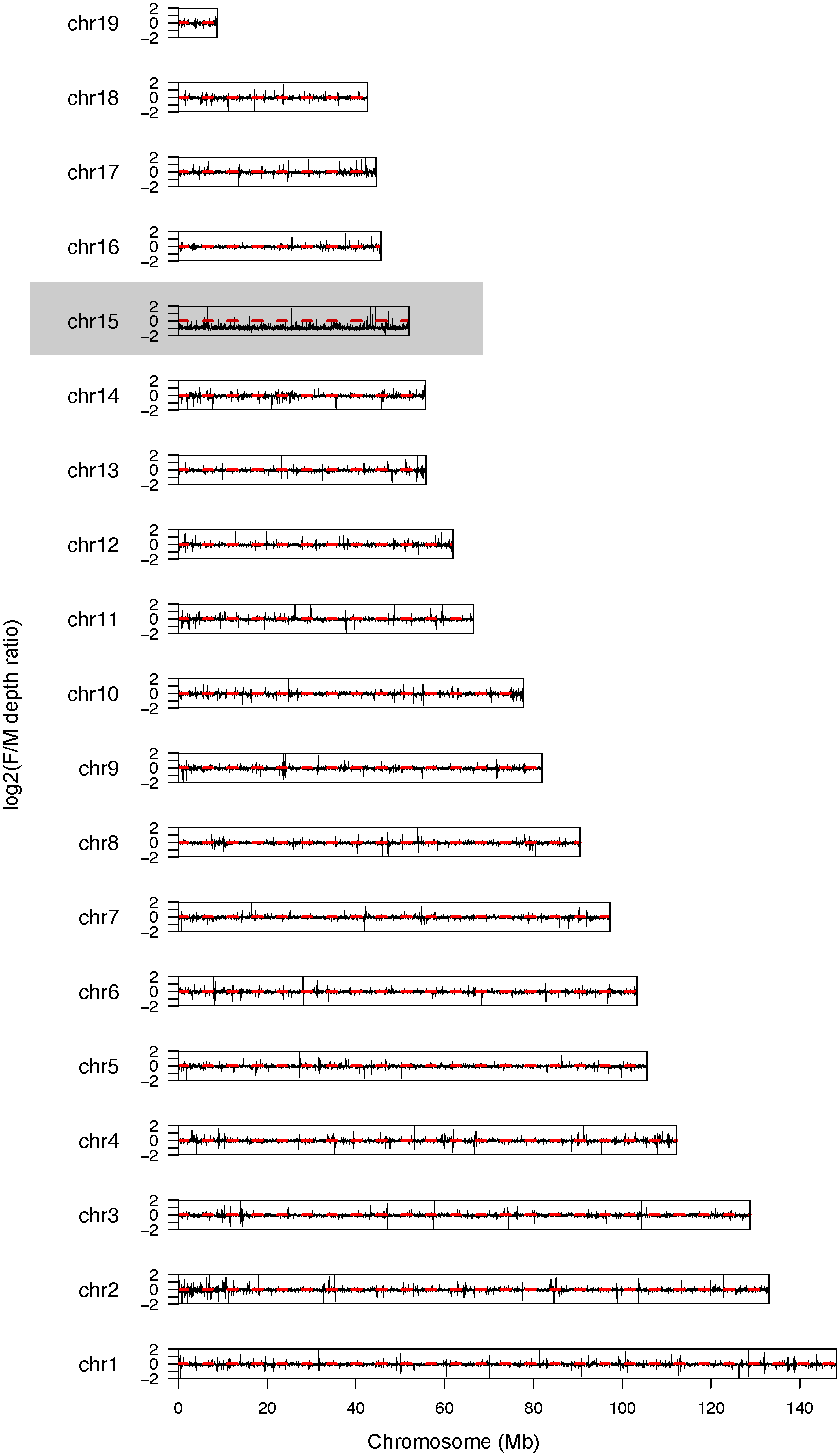
**

**Figure S5.** The female to male depth ratio (log_2_) for each chromosome. The depth was calculated using 100 kb window. The sequence coverage of chromosome 15 in females was roughly half that of males throughout all of the chromosome.


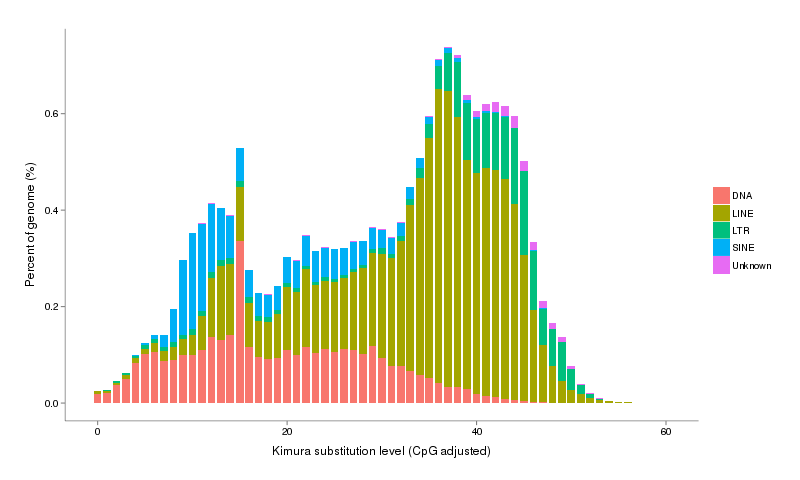


**Figure S6.** Divergence distribution of classified families in Mongolian racerunner genome.


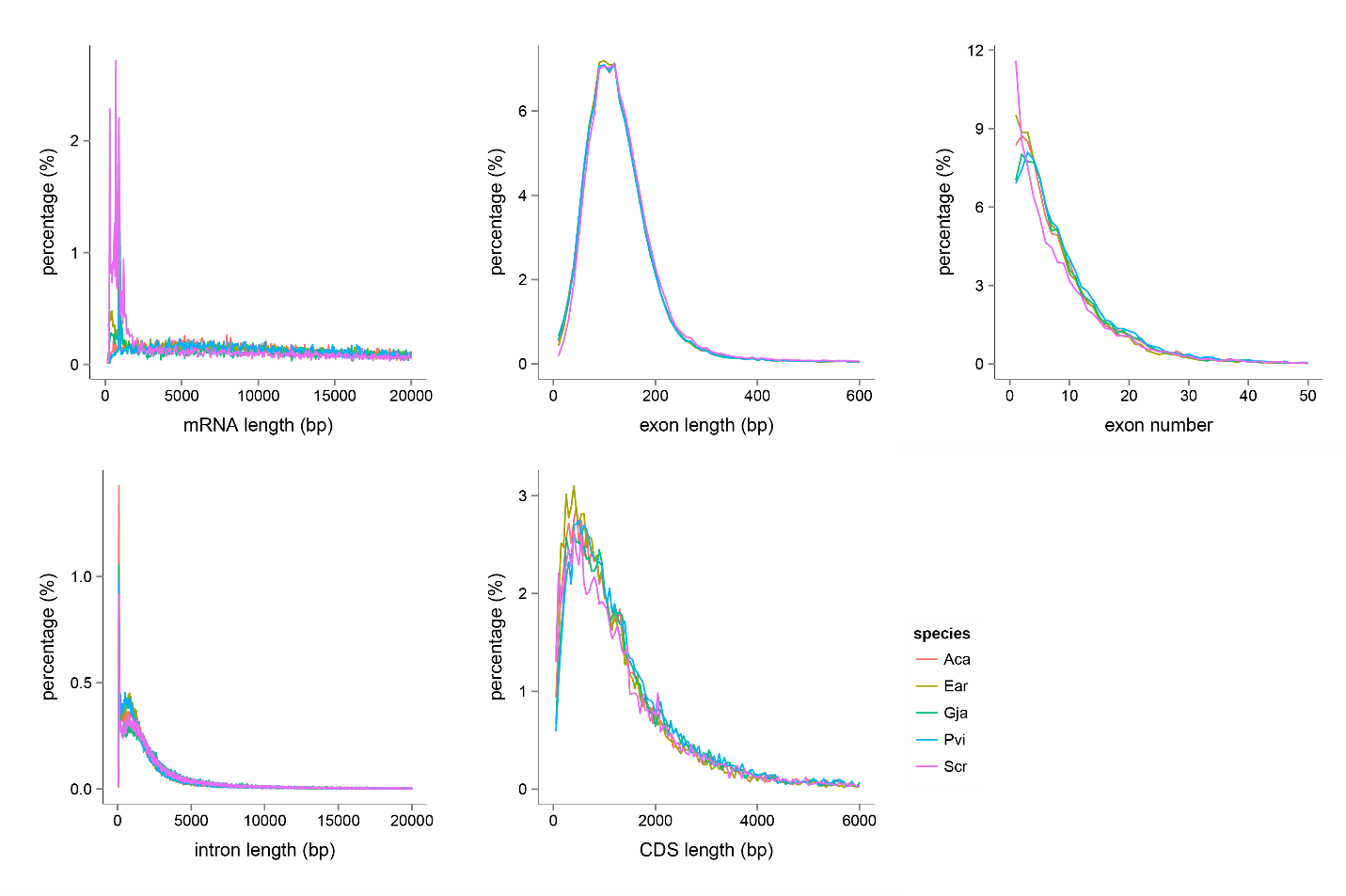


**Figure S7.** A **c**omparison of gene parameters among the five lizard genomes. The gene parameters were similar between the Mongolian racerunner and other lizards, which indicates the high quality gene structure annotation in the Mongolian racerunner genome.

Note: Aca, *Anolis carolinensis*; Ear, *Eremias argus*; Gja, *Gekko japonicas*; Pvi, *Pogona vitticeps*;Scr, *Shinisaurus crocodilurus.*

*
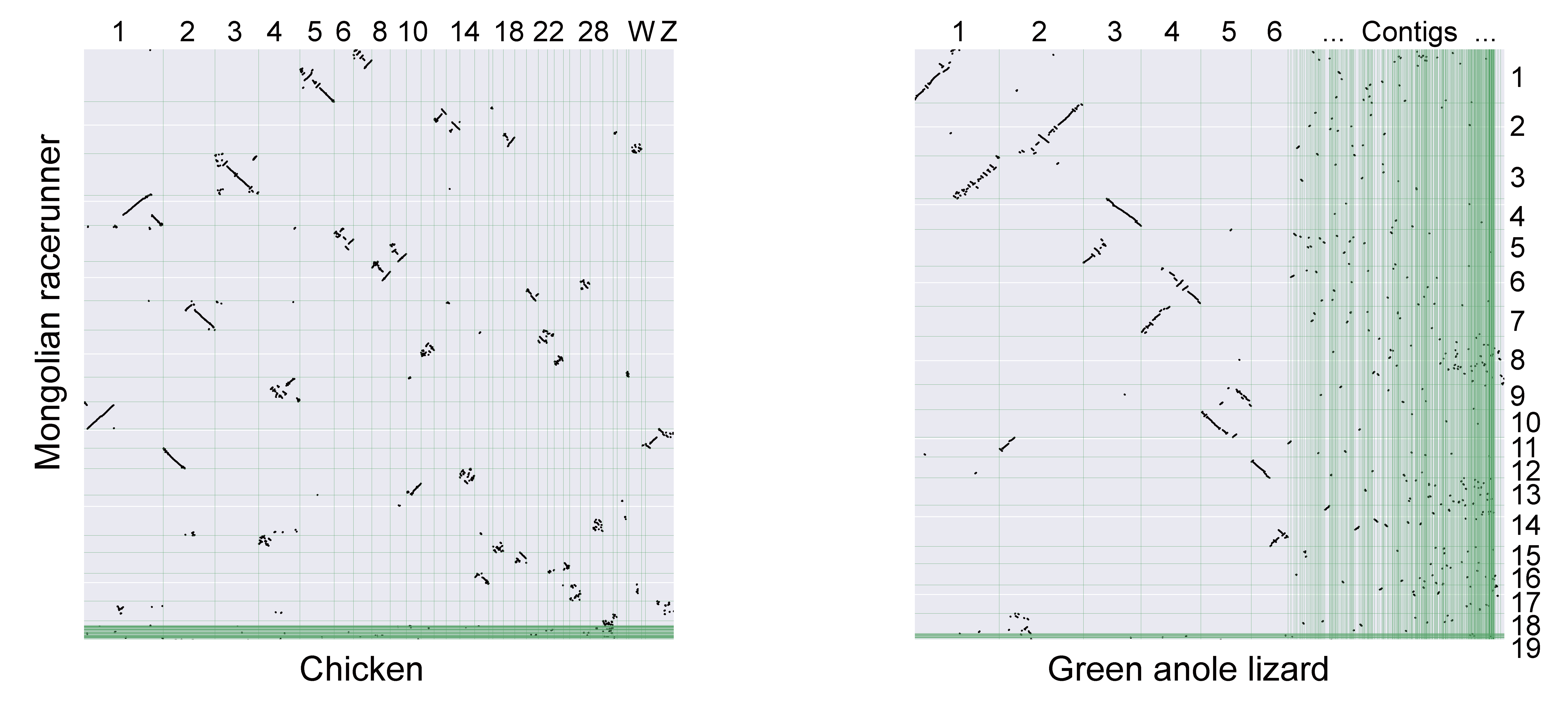
*

**Figure S8.** Synteny between chicken and Mongolian racerunner genomes (left), and green anole lizard and Mongolian racerunner genomes (right).

*
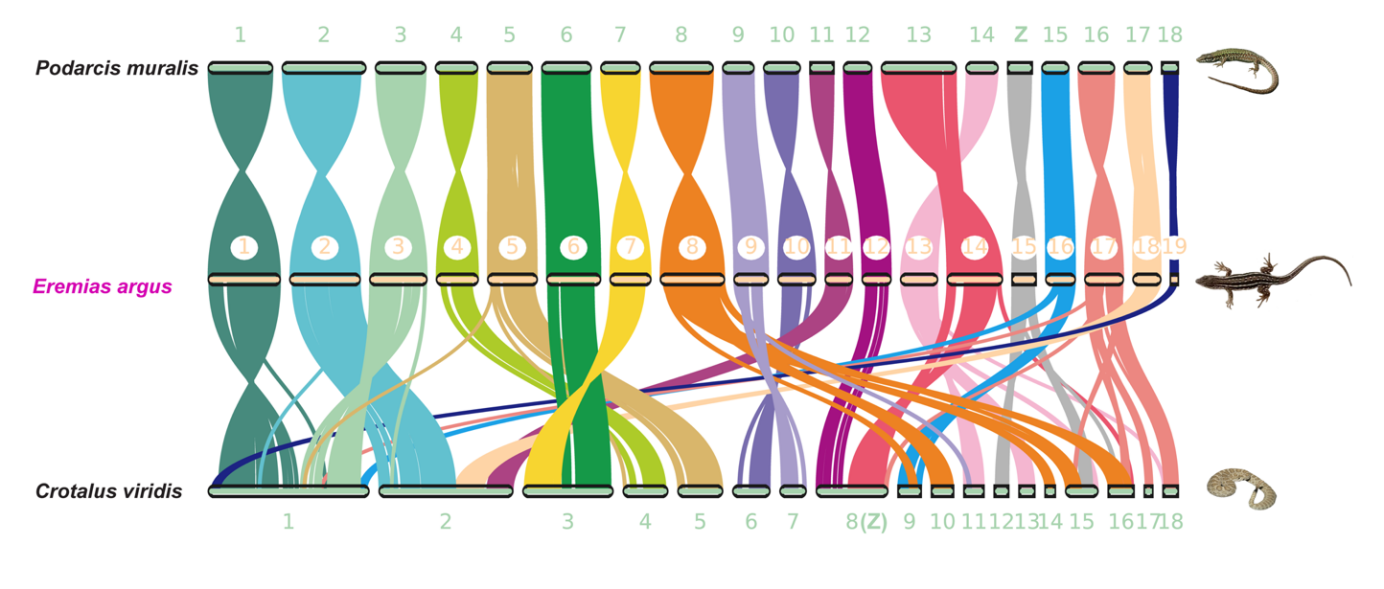
*

**Figure S9.** Comparative synteny of chromosomes among European wall common lizard (*P. muralis*), Mongolian racerunner and rattlesnake (*C. viridis*) genomes.


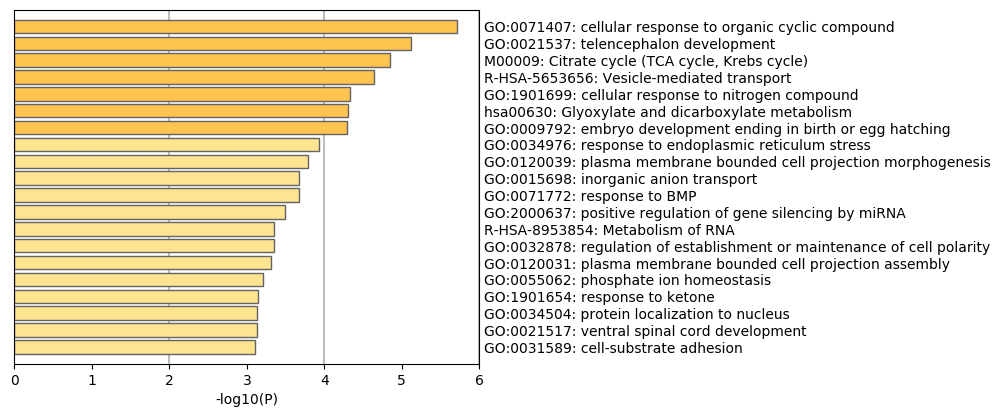


**Figure S10.** The top 20 functional enrichment results of genes located at rattlesnake genome chromosome breakpoint regions using Metascape.


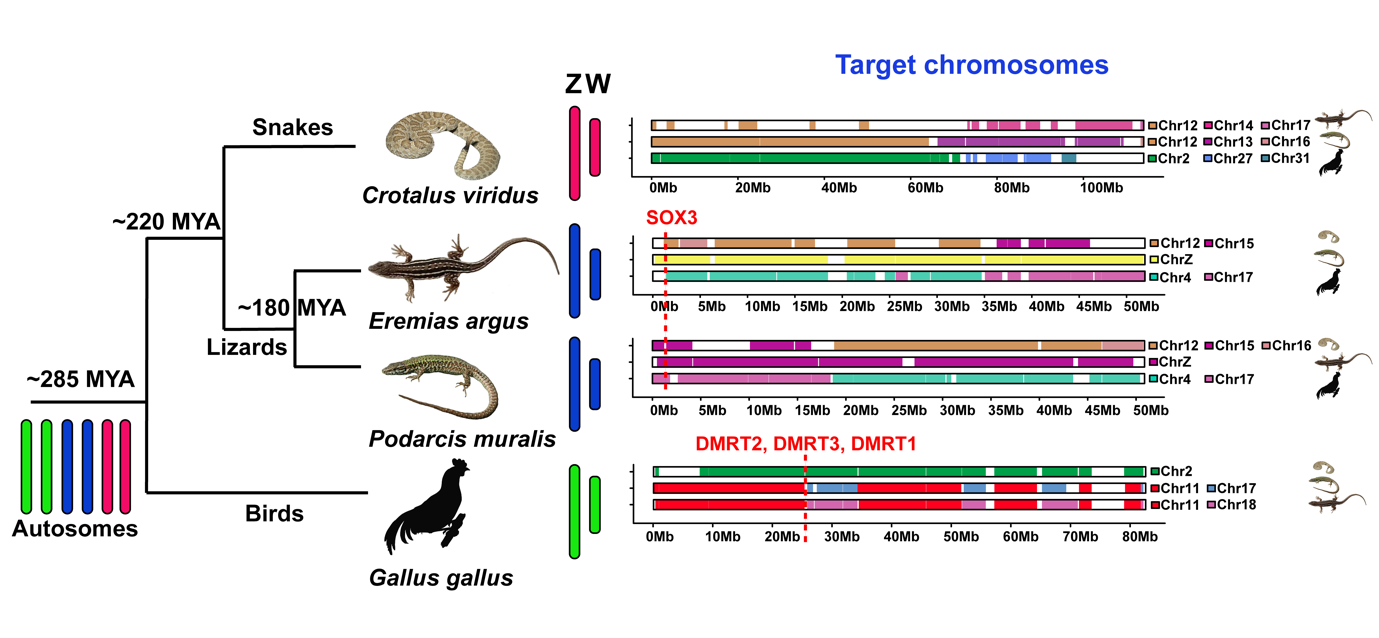


**Figure S11.** Synteny of the Z chromosome among four species. The chromosome painting depicts synteny between Z chromosome of one species and the whole chromosomes of the other three species, respectively. Especially, *SOX3* gene, as a sex determination gene for mammals [2], was located at the chromosome Z of Mongolian racerunner. *DMRT1*, *DMRT2*, and *DMRT3*, as sex determination genes for birds, were located at the chromosome 18 of Mongolian racerunner. These four genes were also located at the chromosome breakpoint regions.


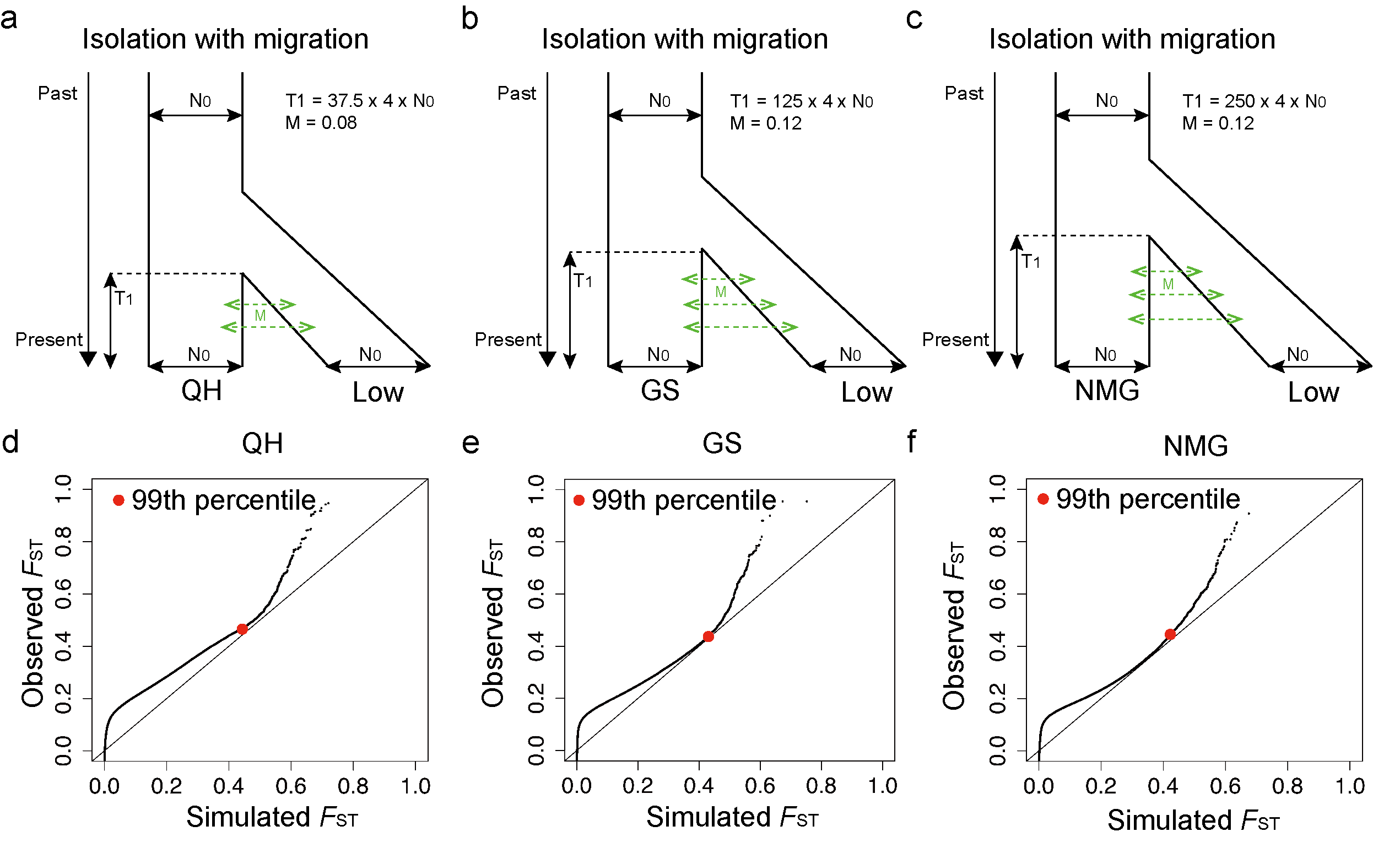


**Figure S12.** The isolation with migration (IWM) models of species formation used for coalescent simulations and quantile-quantile plots comparing the distributions of observed *F*_ST_ values with simulated *F*_ST_ values. The position of the 99^th^ percentile of observed and simulated *F*_ST_ is indicated in red. Similar patterns are observed for NMG, GS and QH simulations indicating approximately the top 1% of observed *F*_ST_ are higher than corresponding simulated *F*_ST_.


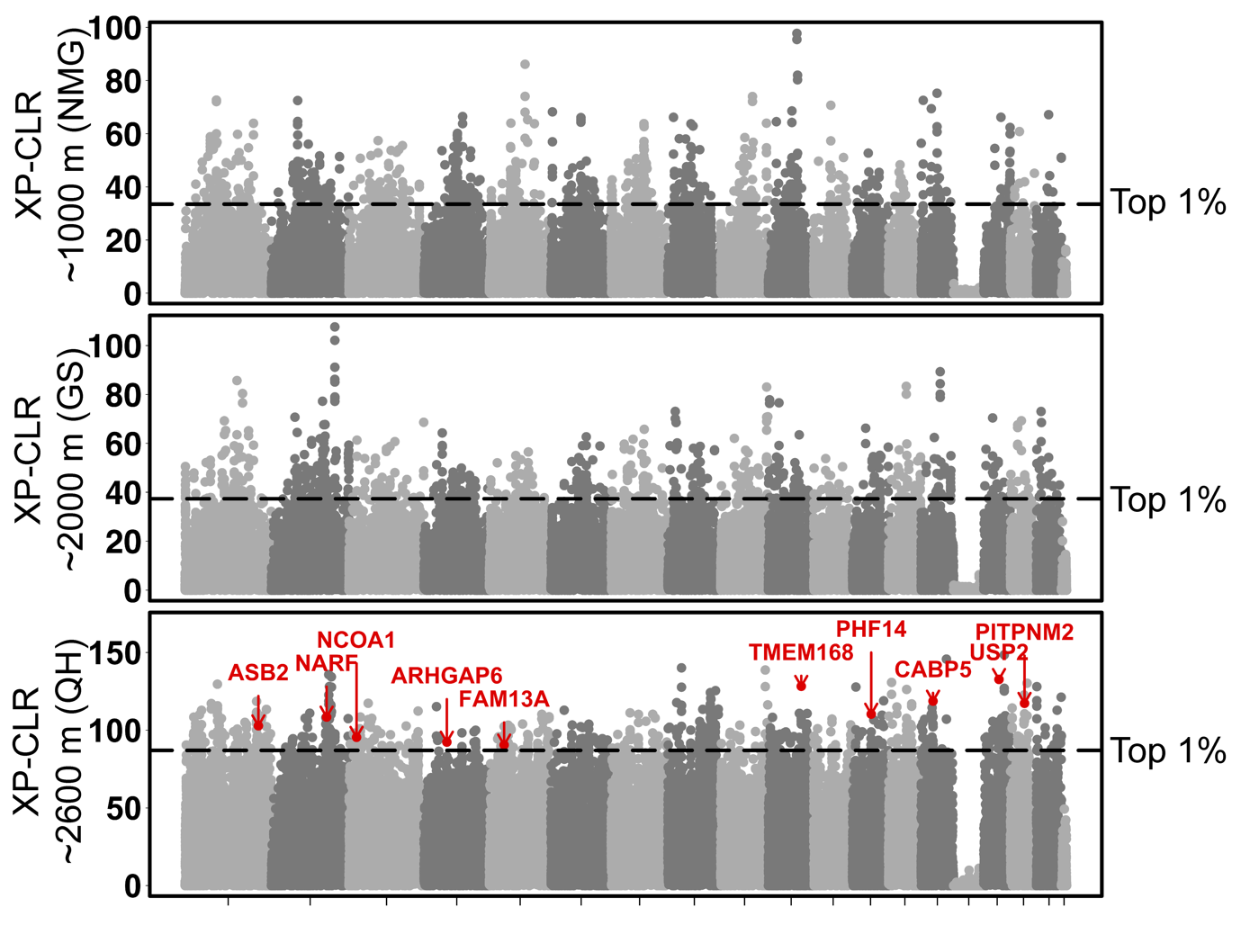


**Figure S13.** This figure showed the distribution of XP-CLR values which calculated in 40-kb window and 20-kb step between high and low altitude populations. The dark dotted line showed the top 1% XP-CLR of values distribution. The pairwises for XP-CLR are the same to *F*_ST_ and log2(*θ*_π_ ratio) analysis: the ~1000 m (NMG) population compared with three low populations (HB,HEB,HN: altitude under ~300m); the ~2000 m (GS) population compared with three low populations and NMG population; the ~2600 m (QH) population compared with three low populations, NMG population and GS population. The genes that pointed out here functioned by responding to hypoxia or UV, which are important for high-altitude adaptation of lizards.


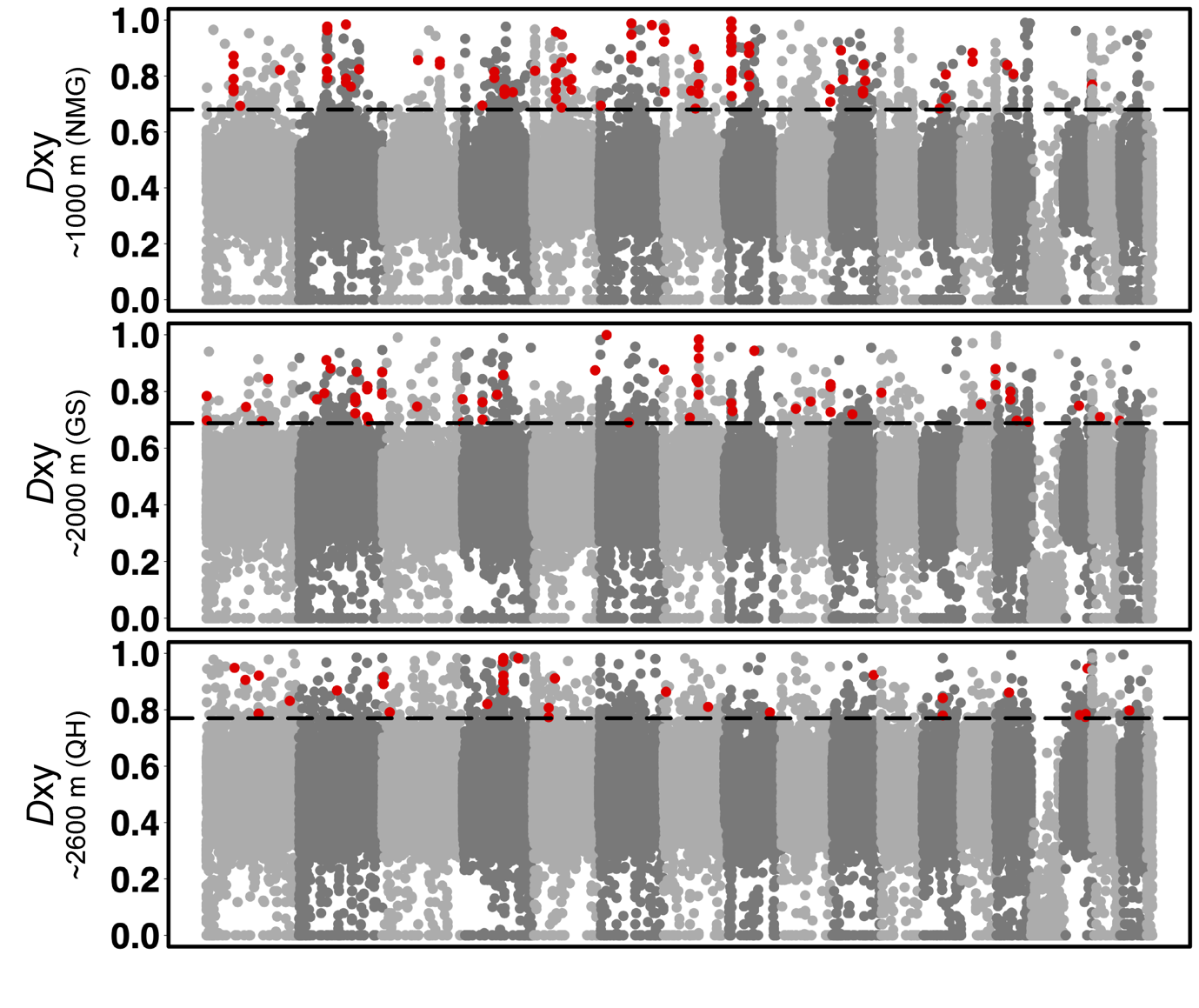


**Figure S14.** This figure showed the distribution of absolute divergence (*D*xy) values which calculated in 40-kb window and 20-kb step between high and low altitude populations. The dark dotted line showed the top 1% *D*xy of values distribution. The red points displayed the top 1% *F*_ST_ and log2(*θ*_π_ ratio) outlier regions. The pairwise for *D*xy are the same as *F*_ST_ and log2(*θ*_π_ ratio) analysis (See the legend of Figure S19).


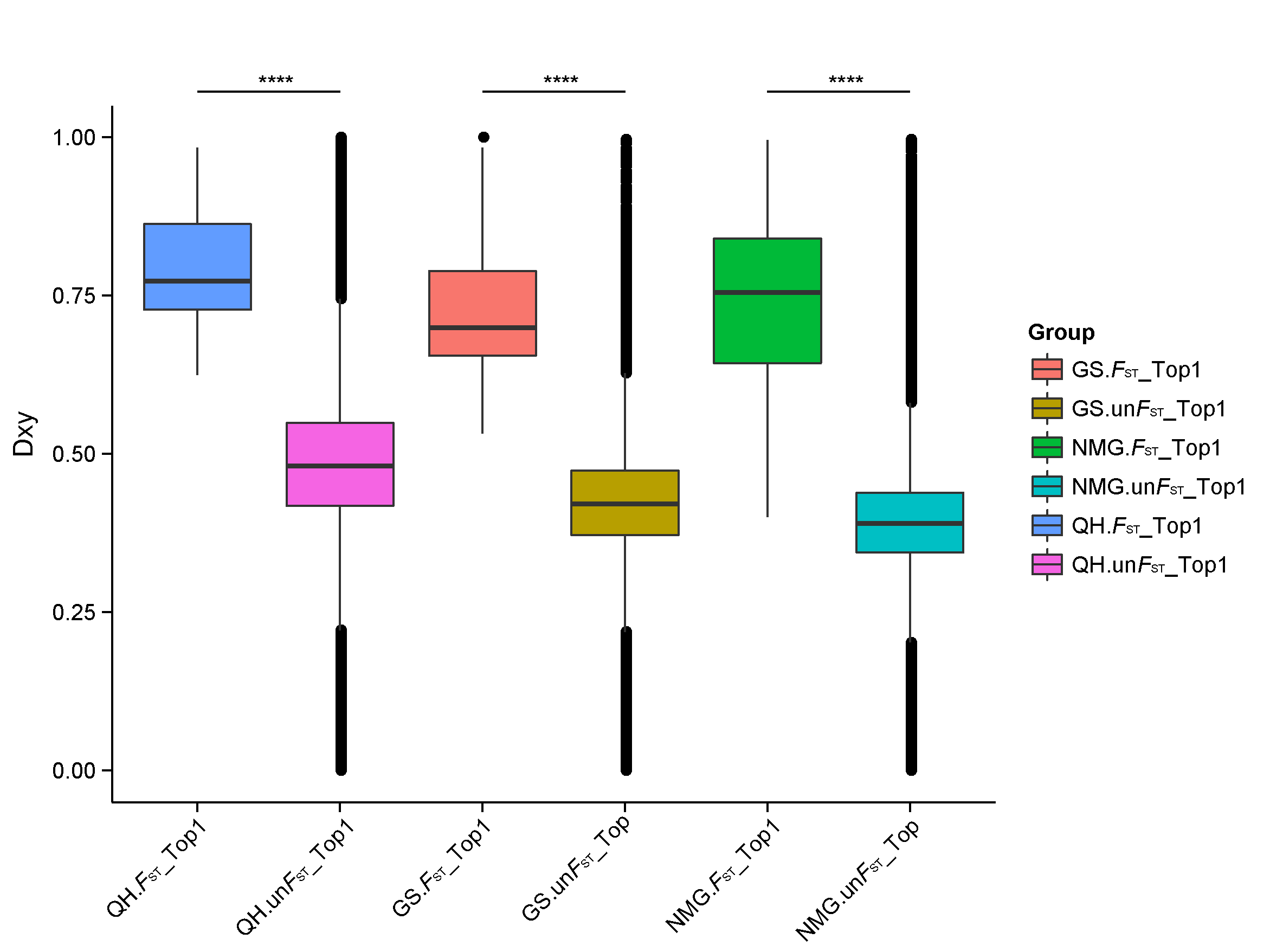


**Figure S15.** The absolute divergence (*D*xy) comparison between the top 1% *F*_ST_ and log2(*θ*_π_ ratio) regions and the rest regions (****, *P* < 0.0001, Wilcoxon signed-rank test).


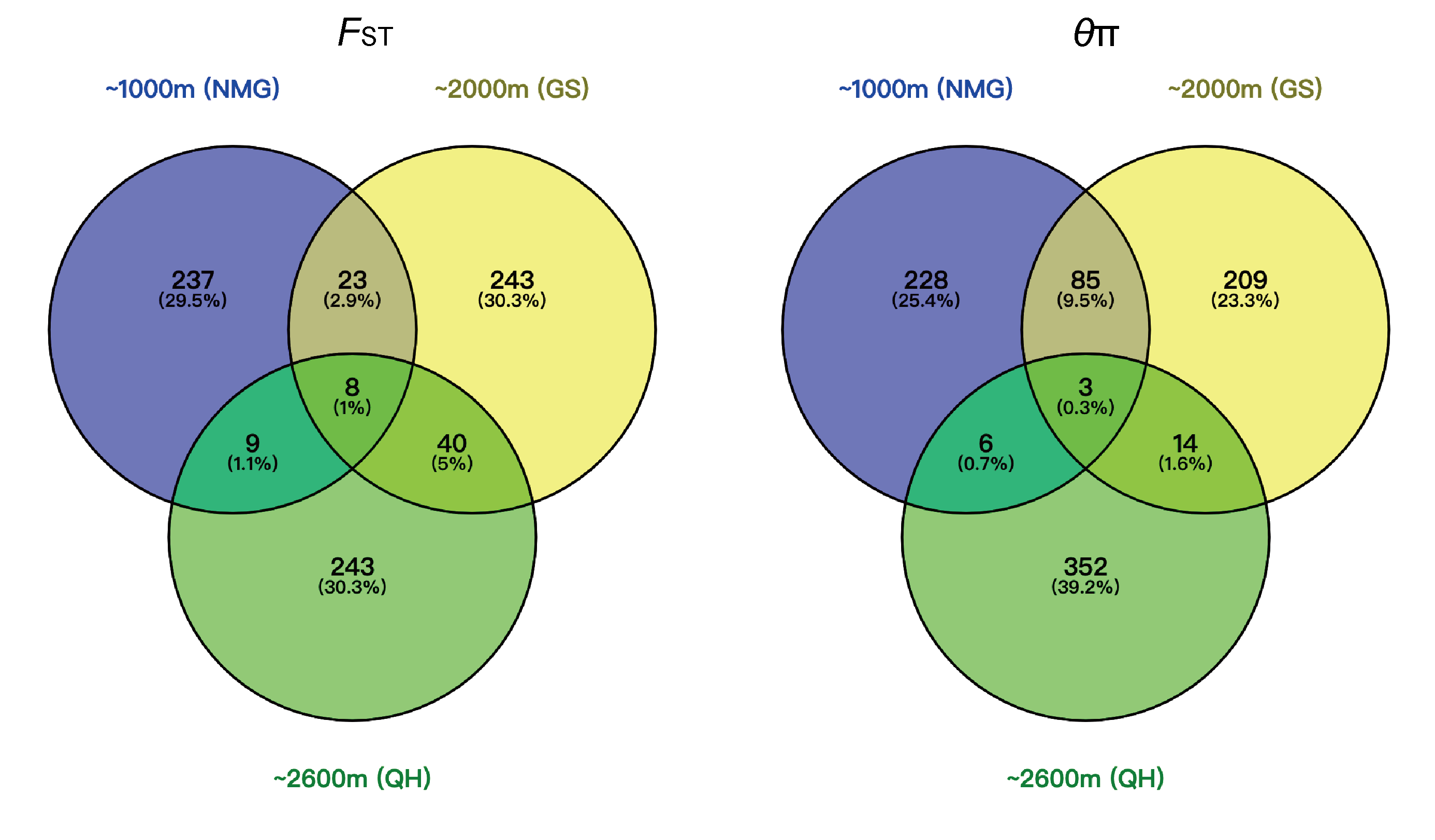


**Figure S16.** Venn plots were shown the global overlaps of the top 1% candidate loci between NMG/GS/QH contrasts from *F*_ST_-based and *θ*_π_-based, respectively.


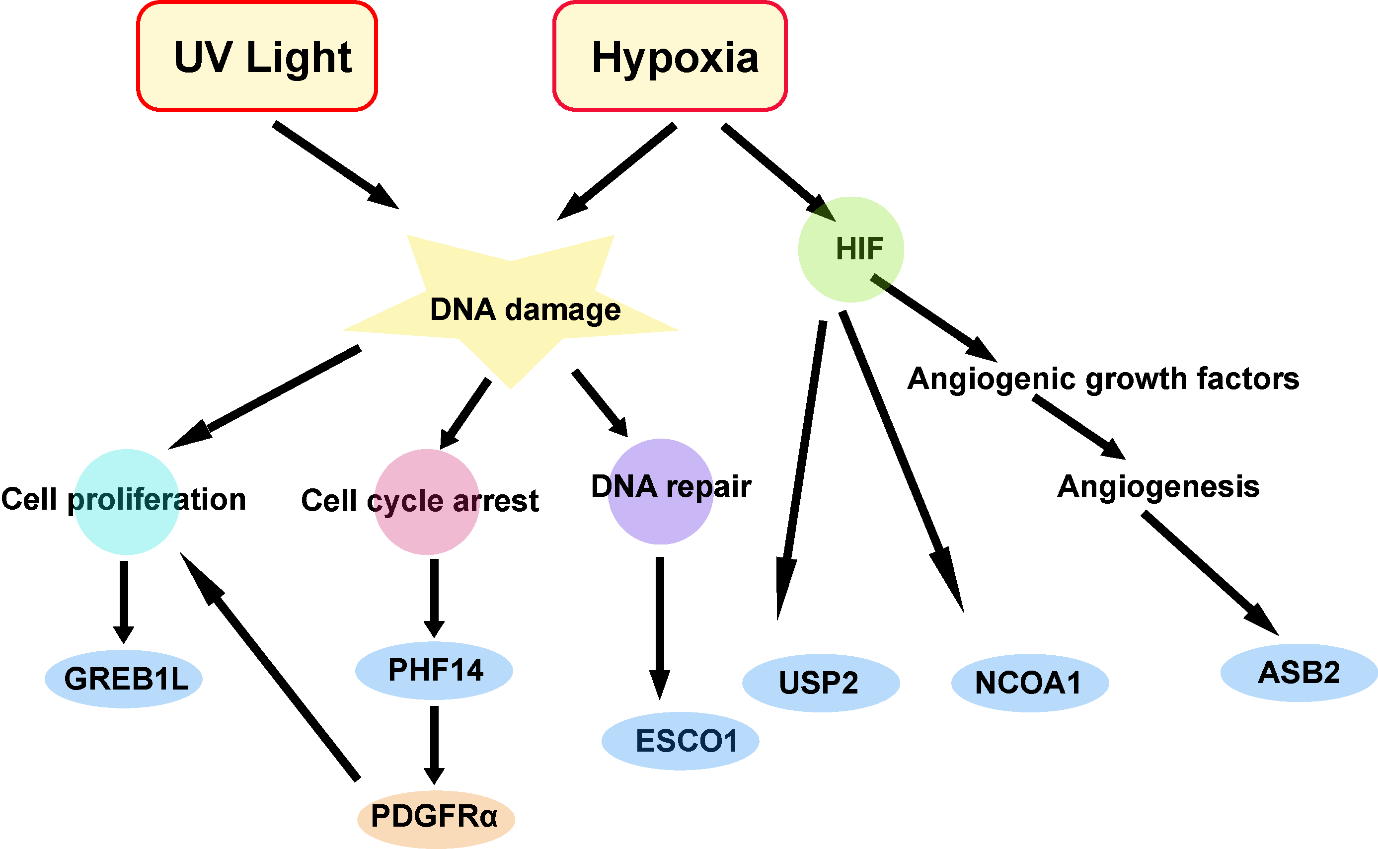


**Figure S17.** A concise map of positively selected genes labeled with blue that may relate to hypoxia and UV light response in the QH population of Mongolian racerunner.


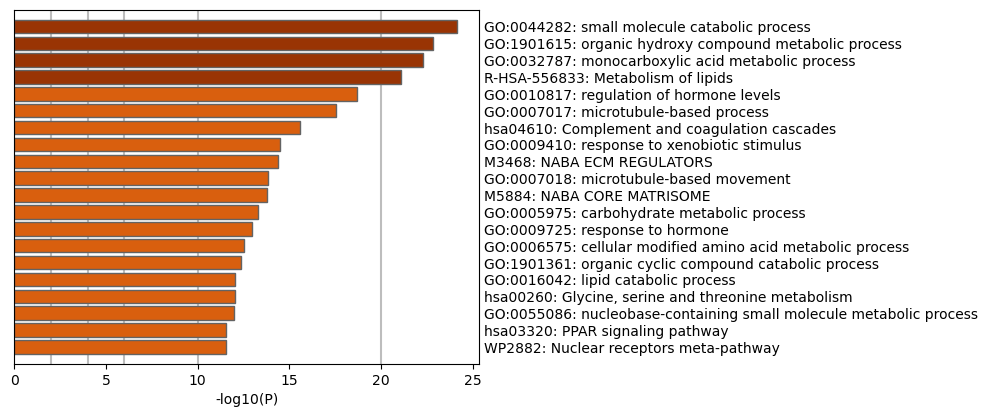


**Figure S18.** The functional enrichment results of differentially expressed genes (DEGs) using Metascape.


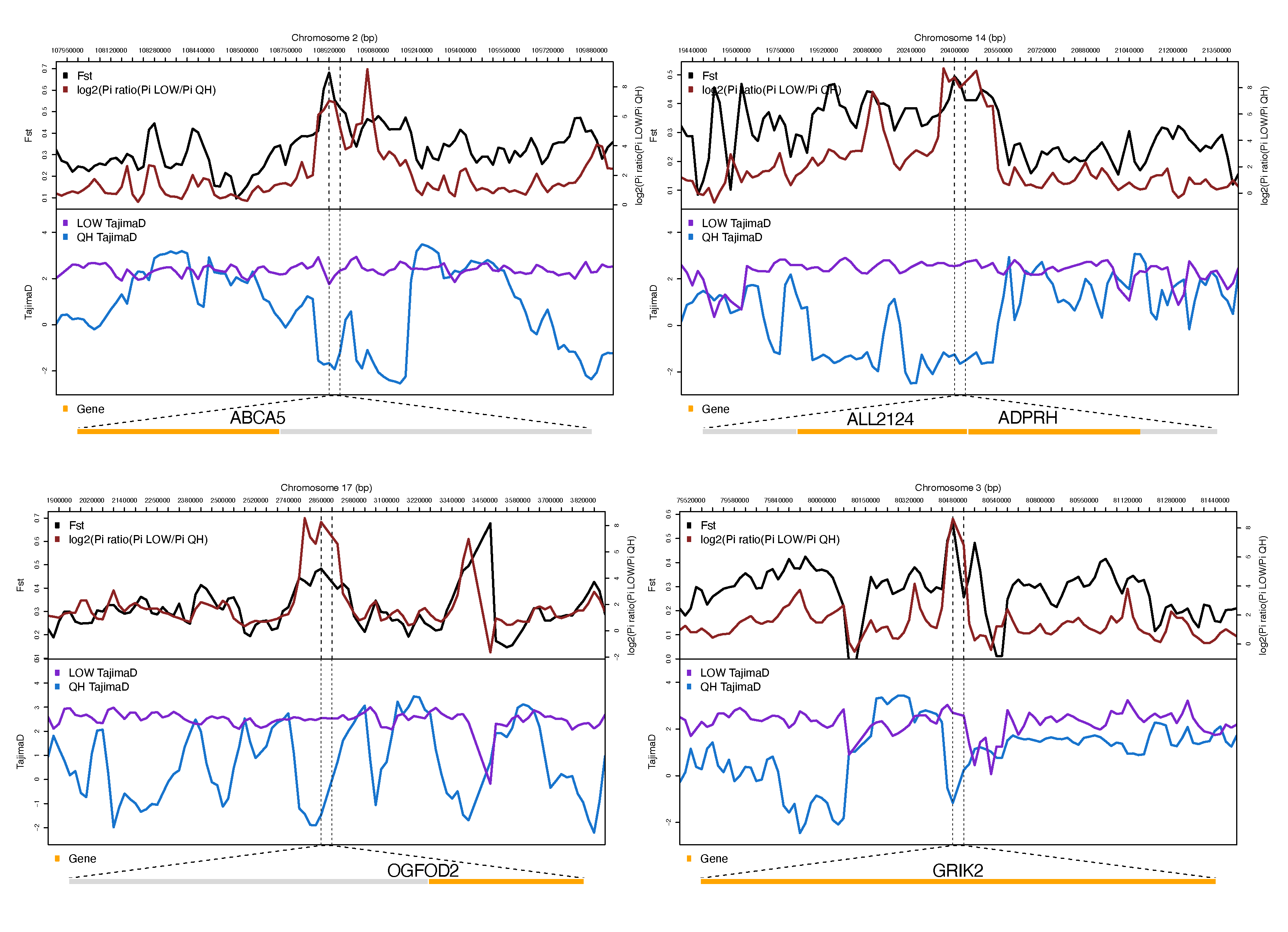


**Figure S19.** The *F*_ST_ and *θ*_π_, and Tajima’*D* for the upstream and downstream 1Mb genome regions of these five genes were calculated in 40kb window. The results showed that these five genes were in higher *F*_ST_ and *θ*_π_, and lower Tajima’*D* genome regions in high-altitude (QH) population compared to low altitude population.


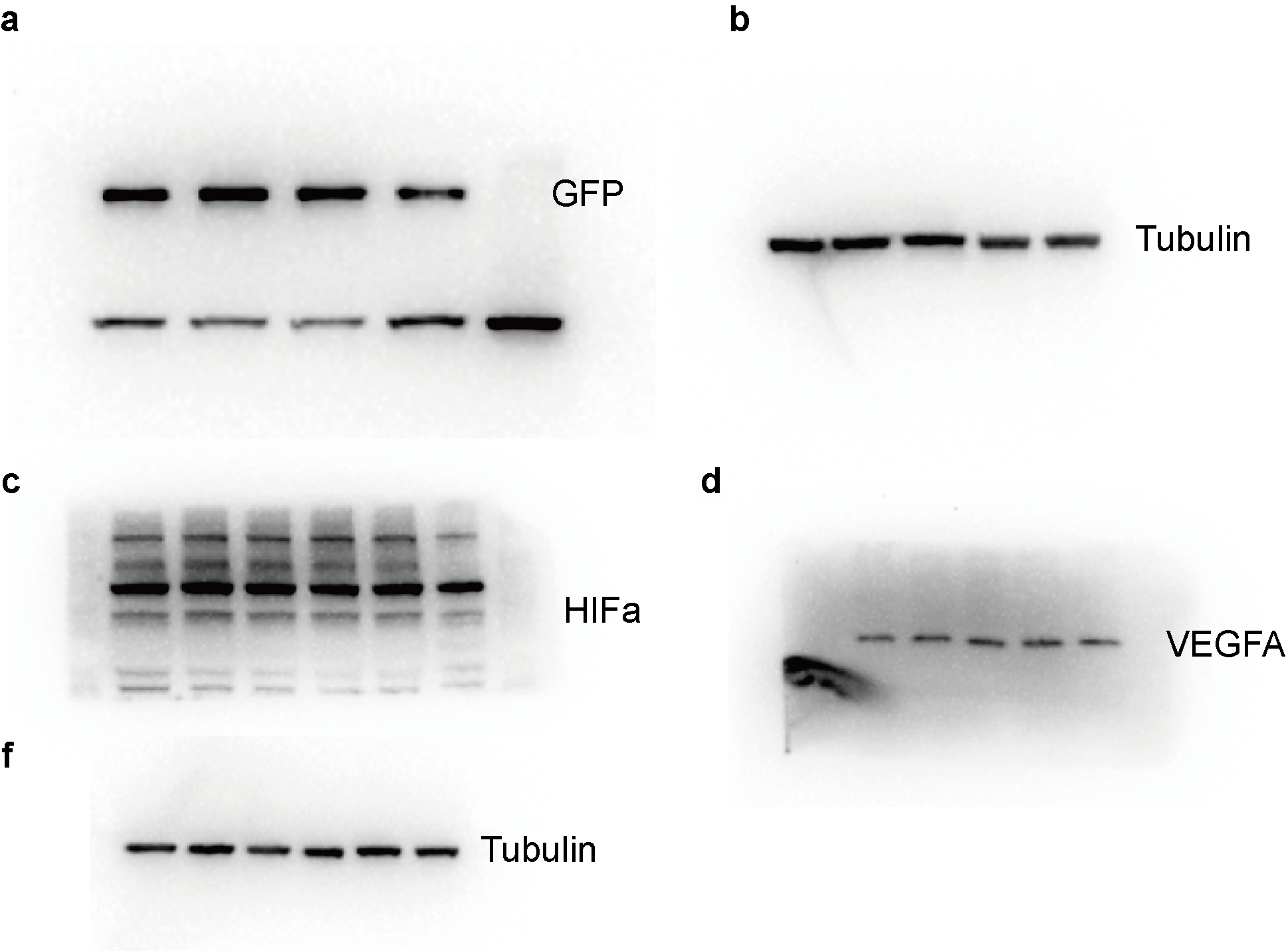


**Figure S20.** The original and uncropped gels for cell experiments included GFP, Tubulin, HIFa and VEGFA.
