# Supplementary material for: Chromosome-level genome assembly and population genomics of Mongolian racerunner (Eremias argus) provide insights into high-altitude adaptation in lizards
Source: BMC Biol. 2023 Feb 20;21:40. doi: 10.1186/s12915-023-01535-z (PMC9942394; doi:10.1186/s12915-023-01535-z)
Supplement: Supplementary file 2 — Additional file 2: Table S1. Reads and libraries used in de novo Mongolian racerunner genome sequencing. Table S2. Assembly statistics of Mongolian racerunner genome. Table S3. The CEGMA (Core Eukaryotic Genes Mapping Approach) result of Mongolian racerunner genome. Table S4. The BUSCO (Benchmarking Universal Single-Copy Orthologs) result of Mongolian racerunner genome. Table S5. The statistics on genome coverage and depth of Mongolian racerunner genome. Table S6. The statistics of repeat sequences in Mongolian racerunner genome. Table S7. Gene prediction of Mongolian racerunner (Eremias argus) genome. Table S8. The statistics on functional gene annotation of Mongolian racerunner genome. Table S9. Geographical position and sex of sampled Mongolian racerunner individuals. Table S10. Mapping statistics of re-sequenced individuals. The genome-wide resequencing data for 61 samples were mapped to the de novo Mongolian racerunner genome. Table S11. Summary of SNPs in 61 re-sequenced individuals of Mongolian racerunner. Table S12. The top 1% genes under select sweep region in the Inner-Mongolia (NMG) population. Table S13. The top 1% genes under select sweep region in the GanSu (GS) population. Table S14. The top 1% genes under select sweep region in the Qinghai (QH) population. Table S15. Biological Process (BP) GO term enrichment results of selected genes in the NMG populations. Table S16. KEGG enrichment results of selected genes in the NMG population. Table S17. Biological Process (BP) GO term enrichment results of selected genes in the GS populations. Table S18. KEGG enrichment results of selected genes in the GS populations. Table S19. Biological Process (BP) GO term enrichment results of selected genes in the QH populations. Table S20. KEGG enrichment results of selected genes in the QH populations. Table S21. The primers for PCR. [file 12915_2023_1535_MOESM2_ESM.docx]

**Chromosome-level genome assembly and population genomics of Mongolian racerunner (*Eremias argus*) provide insights into high-altitude adaptation in lizards**

Weiming Li^1,2,5^, Juan Du^1,2,5^, Lingyun Yang^3,5^, Qiqi Liang^3^, Mengyuan Yang^1,2^, Xuming Zhou*^,1^, Weiguo Du*^,1,4^

^1^Key Laboratory of Animal Ecology and Conservation Biology, Institute of Zoology, Chinese Academy of Sciences, Beijing 100101, China.

^2^University of Chinese Academic of Sciences, China.

^3^Novogene Bioinformatics Institute, 100083 Beijing, China.

^4^Center for Excellence in Animal Evolution and Genetics, Chinese Academy of Sciences, Kunming 650223, China.

^5^These authors contributed equally.

*Correspondence and requests for materials should be addressed to W.D. (E-mail: duweiguo@ioz.ac.cn) or to X.Z. (E-mail: zhouxuming@ioz.ac.cn).

**Supplementary Tables**

**Table S1.** Reads and libraries used in *de novo* Mongolian racerunner genome sequencing.

| **Pair-end libraries** | **Insert size** | **Read length (bp)** | **Total data (G)** |
| --- | --- | --- | --- |
| Pacbio reads | 6.50 Kb | -- | 187.94 |
| Illumina | 350 bp | 150 | 106.44 |
| 10X Genomics | -- | 150 | 194.14 |
| Hi-C | -- | 150 | 233.50 |
| Total | -- | -- | 722.02 |

**Table S2.** Assembly statistics of Mongolian racerunner genome.

| **Method** | **Title** | **Total length** | **Total number** | **Average length** | **Max length** | **N50 length** | **N50 number** | **N90 length** | **N90 number** |
| --- | --- | --- | --- | --- | --- | --- | --- | --- | --- |
| Falcon | Contig | 1,750,809,939 | 2,685 | 652,070 | 12,382,159 | 2,796,973 | 181 | 414,640 | 734 |
| Quiver | Contig | 1,756,032,267 | 2,685 | 654,015 | 12,405,543 | 2,806,597 | 181 | 415,426 | 734 |
| 10X | Contig* | 1,632,399,615 | 1,259 | 1,296,584 | 12,405,543 | 3015371 | 160 | 828,699 | 551 |
|  | Scaffold | 1,635,056,283 | 885 | 1,847,521 | 34,132,755 | 4,436,323 | 109 | 1,286,693 | 371 |
| Pilon | Contig* | 1,631,155,376 | 1,249 | 1,305,969 | 12,400,277 | 3,066,547 | 156 | 827,820 | 546 |
|  | Scaffold | 1,634,012,939 | 307 | 5,322,517 | 202,920,024 | 96,734,774 | 6 | 37,323,541 | 17 |
| Hi-C | Contig* | 1,631,155,376 | 1,249 | 1,305,969 | 12,400,277 | 3,066,547 | 156 | 827,820 | 546 |
|  | Scaffold | 1,631,214,376 | 659 | 2,475,287 | 148,131,413 | 97,224,971 | 7 | 42,694,005 | 18 |

**Note:** Contig* represented Contig after scaffold. The scaffolds with a length of < 100bp were not included in the statistics.

**Table S3.** The CEGMA (Core Eukaryotic Genes Mapping Approach) result of Mongolian racerunner genome.

| **Species** | **Complete** | | **Complete + Partial** | |
| --- | --- | --- | --- | --- |
|  | **#Prots** | **%Completeness** | **#Prots** | **%Completeness** |
| Mongolian racerunner | 226 | 91.13 | 235 | 94.76 |

**Table S4.** The BUSCO (Benchmarking Universal Single-Copy Orthologs) result of Mongolian racerunner genome.

| **Species** | **BUSCO notation assessment results** |
| --- | --- |
| Mongolian racerunner | C:94.39% [S:93.3%, D:1.6%],F:2.9%,M:2.2%, n: Vertebrate |

**Table S5.** The statistics on genome coverage and depth of Mongolian racerunner genome.

|  |  | **Percentage** |
| --- | --- | --- |
| Reads | Mapping rate (%) | 98.65% |
| Assembly | Average sequencing depth | 38.69 |
|  | Coverage | 99.74% |
|  | Coverage at least 4X | 99.62% |
|  | Coverage at least 10X | 99.08% |
|  | Coverage at least 20X | 92.59% |

**Table S6.** The statistics of repeat sequences in Mongolian racerunner genome.

|  | **Denovo+Repbase** | | **TE Proteins** | | **Combined TEs** | |
| --- | --- | --- | --- | --- | --- | --- |
|  | **Length**  **(bp)** | **%in Genome** | **Length**  **(bp)** | **% in Genome** | **Length**  **(bp)** | **% in Genome** |
| DNA | 80,039,001 44991380.165435 7188138 0.264310 0 0.000000 10426577 0.383388 | 4.90 | 9,835,067 | 0.60 | 84,012,688 | 5.14 |
| LINE | 509,892,180 | 31.20 | 106,322,183 | 6.51 | 542,159,307 | 33.18 |
| LTR | 200,931,490 | 12.30 | 33,344,718 | 2.04 | 204,736,973 | 12.53 |
| SINE | 26,315,439 | 1.61 | \ | \ | 26,315,439 | 1.61 |
| Satellite | 1,287,816 | 0.08 | \ | \ | 1,287,816 | 0.08 |
| Simple_repeat | 4,419,229 | 0.27 | \ | \ | 4,419,229 | 0.27 |
| Unknown | 2,596,312 | 0.16 | \ | \ | 2,596,312 | 0.16 |
| Total | 751,704,447 | 46.00 | 149,351,316 | 9.14 | 766,717,352 | 46.92 |

**Table S7.** Gene prediction of Mongolian racerunner (*Eremias argus*) genome.

| **Gene set** | | **Number** | **Average gene length (bp)** | **Average CDS length (bp)** | **Average exons per gene** | **Average exon length (bp)** | **Average intron length (bp)** |
| --- | --- | --- | --- | --- | --- | --- | --- |
| *De novo* | Augustus | 23,104 | 20,943.89 | 1,243.12 | 6.66 | 186.56 | 3,478.56 |
|  | Glimmer | 124,426 | 12,023.25 | 441.37 | 3.71 | 119.09 | 4,279.90 |
|  | SNAP | 46,723 | 58,823.40 | 818.11 | 5.98 | 136.87 | 11,654.14 |
|  | Geneid | 28,146 | 33,271.92 | 1,232.61 | 6.63 | 186.05 | 5,695.81 |
|  | Genscan | 32,903 | 36,059.92 | 1,339.17 | 7.85 | 170.50 | 5,065.36 |
| Homolog | Aca | 31,712 | 13,335.76 | 1,243.31 | 5.14 | 242.01 | 2,922.74 |
|  | Pvi | 30,540 | 13,962.25 | 1,242.62 | 5.53 | 224.82 | 2,809.58 |
|  | Scr | 45,456 | 9,242.28 | 913.61 | 3.77 | 242.17 | 3,003.83 |
|  | Gja | 24,386 | 14,791.57 | 1,209.23 | 5.95 | 203.22 | 2,743.63 |
|  | Pbi | 27,112 | 14,157.90 | 1,155.04 | 5.78 | 203.94 | 2,788.08 |
|  | Pmu | 25,838 | 14,533.60 | 1,269.56 | 4.52 | 219.76 | 2,776.67 |
|  | Tsi | 31,999 | 11,243.04 | 1,086.97 | 4.52 | 240.60 | 2,887.08 |
|  | Dac | 57,223 | 6,614.22 | 901.15 | 3.29 | 274.12 | 2,497.62 |
| RNAseq | Cufflinks | 76,837 | 28,389.68 | 2,992.43 | 7.05 | 424.61 | 4,199.70 |
|  | PASA | 76,594 | 18,742.77 | 995.82 | 5.86 | 170.07 | 3,655.17 |
| EVM | | 23,898 | 22,627.81 | 1,260.64 | 7.46 | 169.03 | 3,308.53 |
| PASA-update | | 23,527 | 25,642.47 | 1,314.22 | 7.74 | 169.80 | 3,609.67 |
| Final set | | 20,107 | 28,715.15 | 1,431.16 | 8.63 | 165.74 | 3,573.59 |

**Note:** *Eremias argus* (Ear),  *Anolis carolinensis* (Aca), *Pogona vitticeps* (Pvi), *Shinisaurus crocodilurus* (Scr), *Gekko japonicas* (Gja), *Python molurus bivittatus* (Pbi), *Protobothrops mucrosquamatus* (Pmu), *Thamnophis sirtalis* (Tsi), *Deinagkistrodon acutus* (Dac).

**Table S8.** The statistics on functional gene annotation of Mongolian racerunner genome.

|  | **Number** | **Percent (%)** |
| --- | --- | --- |
| Total | 20,107 | -- |
| Swissprot | 19,042 | 94.70 |
| Nr | 19,617 | 97.56 |
| KEGG | 16,958 | 84.34 |
| InterPro | 19,990 | 99.42 |
| GO | 18,658 | 92.79 |
| Pfam | 16,503 | 82.08 |
| Annotated | 20,043 | 99.68 |
| Unannotated | 58 | 0.29 |

**Table S9.** Geographical position and sex of sampled Mongolian racerunner individuals.

| **NO.** | **Sex** | **Latitude** | **Longitude** | **Altitude** |
| --- | --- | --- | --- | --- |
| NMG1-1 | M | 40.196°N | 111.121°E | 1021m |
| NMG1-2 | F | 40.196°N | 111.121°E | 1022m |
| NMG1-3 | F | 40.196°N | 111.121°E | 1023m |
| NMG3-1 | M | 40.217°N | 111.094°E | 1020m |
| NMG3-2 | F | 40.217°N | 111.094°E | 1021m |
| NMG3-3 | M | 40.217°N | 111.094°E | 1022m |
| NMG5-1 | F | 40.208°N | 110.729°E | 1020m |
| NMG4-2 | F | 40.208°N | 110.729°E | 1021m |
| NMG4-3 | M | 40.208°N | 110.729°E | 1022m |
| NMG2-1 | F | 40.214°N | 111.126°E | 1020m |
| NMG2-2 | M | 40.214°N | 111.126°E | 1021m |
| QH1-1 | M | 36.191°N | 110.707°E | 2600m |
| QH1-2 | F | 36.191°N | 110.707°E | 2601m |
| QH1-3 | F | 36.191°N | 110.707°E | 2602m |
| QH1-4 | M | 36.191°N | 110.707°E | 2603m |
| QH2-1 | M | 36.146°N | 110.707°E | 2585m |
| QH2-2 | F | 36.146°N | 110.707°E | 2586m |
| QH2-3 | M | 36.146°N | 110.707°E | 2587m |
| QH2-4 | F | 36.146°N | 110.707°E | 2588m |
| QH3-1 | F | 36.2648°N | 100.6022°E | 2585m |
| QH3-2 | M | 36.2648°N | 100.6022°E | 2585m |
| HB1-1 | F | 36.9797°N | 114.4424°E | 80.49m |
| HB1-2 | M | 36.9797°N | 114.4432°E | 80.07m |
| HB1-3 | F | 36.9797°N | 114.4429°E | 81.45m |
| HB2-1 | M | 36.9799°N | 114.4393°E | 79.32m |
| HB2-2 | M | 36.9797°N | 114.4400°E | 80.12m |
| HB3-1 | M | 36.9781°N | 114.4358°E | 63.84m |
| HB3-2 | F | 36.9788°N | 114.4355°E | 68.59m |
| HB4-1 | F | 36.9734°N | 114.4542°E | 81m |
| HB4-2 | M | 36.9734°N | 114.4542°E | 82m |
| HEB1_1 | F | 45.41°N | 126.15°E | 119m |
| HEB1_2 | M | 45.41°N | 126.15°E | 120m |
| HEB1_3 | F | 45.41°N | 126.15°E | 121m |
| HEB1_4 | F | 45.41°N | 126.15°E | 122m |
| HEB1_5 | F | 45.41°N | 126.15°E | 123m |
| HEB1_6 | F | 45.41°N | 126.15°E | 124m |
| HEB1_7 | M | 45.41°N | 126.15°E | 125m |
| HEB2_1 | M | 45.48°N | 126.37°E | 117m |
| HEB3_1 | M | 45.48°N | 126.38°E | 115m |
| HEB3_2 | M | 45.48°N | 126.38°E | 115m |
| HN1-1 | M | 34.5033°N | 112.5740°E | 300m |
| HN1-2 | F | 34.5033°N | 112.5741°E | 305m |
| HN2-1 | F | 34.4915°N | 112.5924°E | 284m |
| HN2-2 | F | 34.4915°N | 112.5925°E | 285m |
| HN2-3 | M | 34.4915°N | 112.5926°E | 285m |
| HN2-4 | F | 34.4915°N | 112.5927°E | 284m |
| HN2-5 | F | 34.4915°N | 112.5928°E | 285m |
| HN3-1 | M | 34.4921°N | 112.5927°E | 260m |
| HN4-1 | M | 34.4810°N | 112.5634°E | 320m |
| HN4-2 | F | 34.4810°N | 112.5635°E | 320m |
| GS1-1 | M | 37.16999°N | 103.87028°E | 1909m |
| GS1-2 | F | 37.1685°N | 103.8693°E | 1918.67m |
| GS1-3 | F | 37.1680°N | 103.8697°E | 1923.41m |
| GS2-1 | M | 37.1486°N | 103.8521°E | 2049.56m |
| GS2-2 | F | 37.1546°N | 103.8604°E | 1997.67m |
| GS3-1 | F | 37.1586°N | 103.8638°E | 1972.25m |
| GS3-2 | F | 37.1590°N | 103.8638°E | 1972.25m |
| GS4-1 | M | 37.1348°N | 103.8470°E | 2179m |
| GS4-2 | M | 37.1348°N | 103.8470°E | 2180m |
| GS4-3 | F | 37.1348°N | 103.8470°E | 2181m |
| GS5_1 | F | 37.1348°N | 103.8470°E | 2179m |

**Table S10.** Mapping statistics of re-sequenced individuals*.* The genome-wide resequencing data for 61 samples were mapped to the *de novo* Mongolian racerunner genome.

| **Sample** | **Clean**  **reads** | **Mapped**  **reads** | **Mapping**  **rate** | **Average**  **Depth** | **Coverage**  **1X** | **Coverage**  **4X** |
| --- | --- | --- | --- | --- | --- | --- |
| GS1_1 | 168708958 | 166673745 | 98.79% | 13.99 | 96.82% | 92.87% |
| GS1_2 | 162209598 | 160014530 | 98.65% | 13.38 | 96.73% | 92.24% |
| GS1_3 | 155998654 | 154029014 | 98.74% | 13.38 | 96.51% | 91.85% |
| GS2_1 | 140601124 | 138821439 | 98.73% | 11.86 | 96.38% | 90.85% |
| GS2_2 | 164742294 | 162658234 | 98.73% | 13.6 | 96.77% | 92.35% |
| GS3_1 | 130657950 | 127997317 | 97.96% | 11.01 | 96.40% | 89.81% |
| GS3_2 | 195612546 | 193539752 | 98.94% | 16.03 | 97.05% | 93.74% |
| GS4_1 | 135819046 | 133275691 | 98.13% | 11.4 | 96.58% | 90.75% |
| GS4_2 | 153846556 | 151681752 | 98.59% | 12.86 | 96.67% | 92.14% |
| GS4_3 | 170470704 | 167595938 | 98.31% | 13.99 | 96.94% | 92.66% |
| GS5_1 | 133900854 | 131634643 | 98.31% | 10.96 | 96.41% | 89.56% |
| HB1-1 | 145847996 | 143284327 | 98.24% | 12.04 | 96.75% | 90.53% |
| HB1-2 | 150821026 | 148418269 | 98.41% | 12.15 | 96.68% | 90.98% |
| HB1-3 | 166253182 | 162171846 | 97.55% | 13.2 | 96.96% | 91.76% |
| HB2-1 | 159884090 | 157621748 | 98.59% | 12.79 | 96.81% | 91.64% |
| HB2-2 | 139441656 | 137188385 | 98.38% | 11.46 | 96.62% | 90.28% |
| HB3-1 | 169272638 | 166910751 | 98.60% | 13.39 | 97.01% | 91.94% |
| HB3-2 | 141382916 | 139033298 | 98.34% | 11.47 | 96.60% | 89.73% |
| HB4_1 | 168269510 | 164211794 | 97.59% | 13.62 | 96.96% | 91.78% |
| HB4_2 | 174942594 | 170753889 | 97.61% | 13.74 | 97.13% | 92.36% |
| HEB1_1 | 127895152 | 125558045 | 98.17% | 10.9 | 95.69% | 87.93% |
| HEB1_2 | 132442088 | 129101821 | 97.48% | 11.21 | 96.08% | 88.61% |
| HEB1_3 | 143072980 | 140415145 | 98.14% | 12.05 | 95.95% | 89.63% |
| HEB1_4 | 129234260 | 126755059 | 98.08% | 11.01 | 95.66% | 88.15% |
| HEB1_5 | 139091962 | 135427189 | 97.37% | 11.54 | 95.86% | 88.72% |
| HEB1_6 | 159561384 | 156333004 | 97.98% | 13.16 | 96.24% | 90.71% |
| HEB1_7 | 140452346 | 137909758 | 98.19% | 11.76 | 95.77% | 89.71% |
| HEB2_1 | 154603328 | 151415474 | 97.94% | 12.36 | 95.90% | 90.21% |
| HEB3_1 | 153612720 | 150995724 | 98.30% | 12.79 | 95.88% | 90.61% |
| HEB3_2 | 155087296 | 153166376 | 98.76% | 12.63 | 95.88% | 90.46% |
| HN1_1 | 160423054 | 158321686 | 98.69% | 13.41 | 97.29% | 92.98% |
| HN1_2 | 163855436 | 161851125 | 98.78% | 13.48 | 97.34% | 92.83% |
| HN2_1 | 173352472 | 170752271 | 98.50% | 14.76 | 97.37% | 93.37% |
| HN2_2 | 173271518 | 171169832 | 98.79% | 14.03 | 97.31% | 93.28% |
| HN2_3 | 169985014 | 167634596 | 98.62% | 14.03 | 97.30% | 93.26% |
| HN2_4 | 168290980 | 166048098 | 98.67% | 14 | 97.41% | 93.20% |
| HN2_5 | 174407290 | 171866176 | 98.54% | 14.39 | 97.49% | 93.45% |
| HN3_1 | 134100740 | 132287598 | 98.65% | 11.7 | 96.94% | 91.37% |
| HN4_1 | 163608452 | 161457401 | 98.69% | 13.51 | 97.30% | 93.03% |
| HN4_2 | 149594212 | 147724242 | 98.75% | 12.52 | 97.17% | 91.93% |
| NMG1_1 | 155269856 | 153337763 | 98.76% | 12.3 | 97.61% | 93.82% |
| NMG1_2 | 150926834 | 148880227 | 98.64% | 11.79 | 97.51% | 92.80% |
| NMG1_3 | 149524450 | 147855625 | 98.88% | 11.79 | 97.62% | 92.99% |
| NMG2_1 | 138219238 | 136596077 | 98.83% | 11.13 | 97.47% | 92.17% |
| NMG2_2 | 163232936 | 161218186 | 98.77% | 12.73 | 97.61% | 94.12% |
| NMG3_1 | 158820584 | 156606141 | 98.61% | 12.65 | 97.63% | 94.08% |
| NMG3_2 | 137339866 | 134812527 | 98.16% | 10.97 | 97.44% | 91.82% |
| NMG3_3 | 141799548 | 139826225 | 98.61% | 11.31 | 97.54% | 92.82% |
| NMG4_2 | 172031016 | 169782670 | 98.69% | 13.3 | 97.66% | 94.01% |
| NMG4_3 | 150912234 | 148923076 | 98.68% | 11.96 | 97.59% | 93.42% |
| NMG5_1 | 160722764 | 158134633 | 98.39% | 12.81 | 97.96% | 94.28% |
| QH1_1 | 136366742 | 134086478 | 98.33% | 11.11 | 95.85% | 90.05% |
| QH1_2 | 136207008 | 134029122 | 98.40% | 11.09 | 96.01% | 89.70% |
| QH1_3 | 147613522 | 145189547 | 98.36% | 11.96 | 96.08% | 90.59% |
| QH1_4 | 140464730 | 138113749 | 98.33% | 11.48 | 96.01% | 90.57% |
| QH2_1 | 137865740 | 135999338 | 98.65% | 11.21 | 95.91% | 90.20% |
| QH2_2 | 102265190 | 100267784 | 98.05% | 8.84 | 95.27% | 83.70% |
| QH2_3 | 123937938 | 121770977 | 98.25% | 10.2 | 95.69% | 88.54% |
| QH2_4 | 140922248 | 138496533 | 98.28% | 11.5 | 95.88% | 89.85% |
| QH3_1 | 154401992 | 151248940 | 97.96% | 12.39 | 96.22% | 91.06% |
| QH3_2 | 155213844 | 152673392 | 98.36% | 12.39 | 96.05% | 91.24% |

**Table S11.** Summary of SNPs in 61 re-sequenced individuals of Mongolian racerunner.

| **Category** | | **Number of SNPs** |
| --- | --- | --- |
| Upstream | | 70,115 |
| Exonic | Stop gain | 339 |
|  | Stop loss | 80 |
|  | Synonymous | 86,638 |
|  | Non-synonymous | 36,003 |
| Intronic | | 2,441,878 |
| Splicing | | 198 |
| Downstream | | 79,049 |
| upstream/downstream | | 4,238 |
| Intergenic | | 3,505,027 |
| ts | | 4,257,507 |
| tv | | 2,045,738 |
| ts/tv | | 2.081 |
| Total | | 6,303,245 |

**Table S12.** The top 1% genes under select sweep region in the Inner-Mongolia (NMG) population**.** The genes marked in red were detected under strong positive selection using *F*_ST_, *θπ*, and XP-CLR.

| **Gene ID** | **Name** | **Description** |
| --- | --- | --- |
| evm.TU.scaffold_100.10 | *TOPORS* | E3 ubiquitin-protein ligase Topors |
| evm.TU.scaffold_110.5 | *SFRP1* | Secreted frizzled-related protein 1 |
| evm.TU.scaffold_115.1268 | *BHLHE40* | Class E basic helix-loop-helix protein 40 |
| evm.TU.scaffold_115.1423 | *PRRC2C* | Protein PRRC2C |
| evm.TU.scaffold_115.1424 | *FMO1* | Dimethylaniline monooxygenase [N-oxide-forming] 1 |
| evm.TU.scaffold_115.330 | *B4GALT5* | Beta-1,4-galactosyltransferase 5 |
| evm.TU.scaffold_115.748 | *TIE1* | Tyrosine-protein kinase receptor Tie-1 |
| evm.TU.scaffold_115.749 | *MPL* | Thrombopoietin receptor |
| evm.TU.scaffold_115.750 | *CDC20* | Cell division cycle protein 20 homolog |
| evm.TU.scaffold_115.751 | *ELOVL1* | Elongation of very long chain fatty acids protein 1 |
| evm.TU.scaffold_129.209 | *PLEKHA5* | Pleckstrin homology domain-containing family A member 5 |
| evm.TU.scaffold_129.249_evm.TU.scaffold_129.248 | *CECR2* | Cat eye syndrome critical region protein 2 |
| evm.TU.scaffold_129.255 | *CACNA1C* | Voltage-dependent L-type calcium channel subunit alpha-1C |
| evm.TU.scaffold_129.796 | *HMGA2* | High mobility group protein HMGI-C |
| evm.TU.scaffold_129.816 | *PPM1H* | Protein phosphatase 1H |
| evm.TU.scaffold_129.833_evm.TU.scaffold_129.834 | *ARID2* | AT-rich interactive domain-containing protein 2 |
| evm.TU.scaffold_168.1096 | *THBD* | Thrombomodulin |
| evm.TU.scaffold_168.1097 | *CD93* | Complement component C1q receptor |
| evm.TU.scaffold_168.483 | *YTHDF3* | YTH domain-containing family protein 3 |
| evm.TU.scaffold_168.484 | *RNF4* | E3 ubiquitin-protein ligase RNF4 |
| evm.TU.scaffold_168.506 | *PLAG1* | Zinc finger protein PLAG1 |
| evm.TU.scaffold_168.515 | *TMEM68* | Transmembrane protein 68 |
| evm.TU.scaffold_168.640 | *NDUFV2* | NADH dehydrogenase [ubiquinone] flavoprotein 2, mitochondrial |
| evm.TU.scaffold_168.641 | *ANKRD12* | Ankyrin repeat domain-containing protein 12 |
| evm.TU.scaffold_195.1685_evm.TU.scaffold_195.1686 | *SPTLC2* | Serine palmitoyltransferase 2 |
| evm.TU.scaffold_195.558 | *ACMSD* | 2-amino-3-carboxymuconate-6-semialdehyde decarboxylase |
| evm.TU.scaffold_195.559 | *TMEM163* | Transmembrane protein 163 |
| evm.TU.scaffold_195.632 | *PTPN4* | Tyrosine-protein phosphatase non-receptor type 4 |
| evm.TU.scaffold_195.691 | *RNF25* | E3 ubiquitin-protein ligase RNF25 |
| evm.TU.scaffold_195.692 | *STK36* | Serine/threonine-protein kinase 36 |
| evm.TU.scaffold_195.694 | *FAM134A* | Protein FAM134A |
| evm.TU.scaffold_197.211 | *TBC1D8* | TBC1 domain family member 8 |
| evm.TU.scaffold_197.213 | *RPL31* | 60S ribosomal protein L31 |
| evm.TU.scaffold_197.421 | *CASK* | Peripheral plasma membrane protein CASK |
| evm.TU.scaffold_197.541 | *SCAF4* | Splicing factor, arginine/serine-rich 15 |
| evm.TU.scaffold_197.542 | *SOD1* | Superoxide dismutase [Cu-Zn] |
| evm.TU.scaffold_197.605 | *GBE1* | 1,4-alpha-glucan-branching enzyme |
| evm.TU.scaffold_209.883 | *NA* | NA |
| evm.TU.scaffold_212.10 | *OLFM2* | Noelin-2 |
| evm.TU.scaffold_219.1 | *IDO2* | Indoleamine 2,3-dioxygenase 2 |
| evm.TU.scaffold_219.2 | *NA* | NA |
| evm.TU.scaffold_219.3 | *ADAM2* | Disintegrin and metalloproteinase domain-containing protein 2 |
| evm.TU.scaffold_251.40 | *OLFM4* | Olfactomedin-4 |
| evm.TU.scaffold_251.42 | *PCDH8* | Protocadherin-8 |
| evm.TU.scaffold_270.321 | *FAM126A* | Hyccin |
| evm.TU.scaffold_270.323 | *TOMM7* | Mitochondrial import receptor subunit TOM7 homolog |
| evm.TU.scaffold_270.439 | *CFAP69* | Cilia- and flagella-associated protein 69 |
| evm.TU.scaffold_270.440 | *STEAP2* | Metalloreductase STEAP2 |
| evm.TU.scaffold_274.2 | *OPRM1* | Mu-type opioid receptor |
| evm.TU.scaffold_49.2 | *TMEM68* | Transmembrane protein 68 |
| evm.TU.scaffold_49.7 | *XKR4* | XK-related protein 4 |
| evm.TU.scaffold_52.1023 | *ARHGAP26* | Rho GTPase-activating protein 26 |
| evm.TU.scaffold_52.1110 | *DLC1* | Rho GTPase-activating protein 7 |
| evm.TU.scaffold_52.1111 | *RARS* | Arginine--tRNA ligase, cytoplasmic |
| evm.TU.scaffold_52.1113 | *WWC1* | Protein KIBRA |
| evm.TU.scaffold_52.1308 | *TNRC6C* | Trinucleotide repeat-containing gene 6C protein |
| evm.TU.scaffold_52.536 | *NA* | NA |
| evm.TU.scaffold_52.539 | *CACNA2D3* | Voltage-dependent calcium channel subunit alpha-2/delta-3 |
| evm.TU.scaffold_52.646 | *TWF2* | Twinfilin-2 |
| evm.TU.scaffold_52.845 | *FAXDC2* | Fatty acid hydroxylase domain-containing protein 2 |
| evm.TU.scaffold_52.847 | *LARP1* | La-related protein 1 |
| evm.TU.scaffold_80.588 | *ANKRD11* | Ankyrin repeat domain-containing protein 11 |
| evm.TU.scaffold_84.2195 | *SYNJ2* | Synaptojanin-2 |
| evm.TU.scaffold_84.2300 | *NHSL1* | NHS-like protein 1 |
| evm.TU.scaffold_84.2633 | *KCNQ5* | Potassium voltage-gated channel subfamily KQT member 5 |
| evm.TU.scaffold_84.456 | *PTEN* | Phosphatidylinositol 3,4,5-trisphosphate 3-phosphatase and dual-specificity protein phosphatase PTEN |
| evm.TU.scaffold_84.466 | *LIPM* | Lipase member M |
| evm.TU.scaffold_84.471 | *LIPN* | Lipase member N |
| evm.TU.scaffold_84.583 | *DOCK1* | Dedicator of cytokinesis protein 1 |
| evm.TU.scaffold_84.698 | *VTI1A* | Vesicle transport through interaction with t-SNAREs homolog 1A |
| evm.TU.scaffold_84.783 | *MGEA5* | Protein O-GlcNAcase |
| evm.TU.scaffold_84.784 | *KCNIP2* | Kv channel-interacting protein 2 |
| evm.TU.scaffold_84.903 | *STAG1* | Cohesin subunit SA-1 |
| evm.TU.scaffold_89.131 | *NCLN* | Nicalin |
| evm.TU.scaffold_93.509 | *MAPT* | Microtubule-associated protein tau |
| evm.TU.scaffold_93.510 | *SLC25A39* | Solute carrier family 25 member 39 |
| evm.TU.scaffold_93.849 | *SYNGR4* | Synaptogyrin-4 |
| evm.TU.scaffold_93.850 | *KDELR1* | ER lumen protein-retaining receptor 1 |

**Table S13.** The top 1% genes under select sweep region in the GanSu (GS) population**.** The genes marked in red were detected under strong positive selection using *F*_ST_, *θπ*, and XP-CLR.

| **Gene ID** | **Name** | **Description** |
| --- | --- | --- |
| evm.TU.scaffold_115.1282 | *BRINP3* | BMP/retinoic acid-inducible neural-specific protein 3 |
| evm.TU.scaffold_115.811 | *PIK3R1* | Phosphatidylinositol 3-kinase regulatory subunit alpha |
| evm.TU.scaffold_129.33 | *TMPRSS9* | Transmembrane protease serine 9 |
| evm.TU.scaffold_129.34 | *ARSA* | Arylsulfatase A |
| evm.TU.scaffold_129.838 | *TMEM117* | Transmembrane protein 117 |
| evm.TU.scaffold_168.539 | *SPIDR* | DNA repair-scaffolding protein |
| evm.TU.scaffold_171.107 | *FOXO1* | Forkhead box protein O1 |
| evm.TU.scaffold_173.5 | *TJP2* | Tight junction protein ZO-2 |
| evm.TU.scaffold_173.6 | *FAM189A2* | Protein FAM189A2 |
| evm.TU.scaffold_195.1 | *AGAP1* | Arf-GAP with GTPase, ANK repeat and PH domain-containing protein 1 |
| evm.TU.scaffold_195.1063 | *TPH* | Tryptophan 5-hydroxylase |
| evm.TU.scaffold_195.1299 | *NA* | NA |
| evm.TU.scaffold_195.1300 | *GYLTL1B* | Glycosyltransferase-like protein LARGE2 |
| evm.TU.scaffold_195.1303 | *PHF21A* | PHD finger protein 21A |
| evm.TU.scaffold_195.1345 | *FUT8* | Alpha-(1,6)-fucosyltransferase |
| evm.TU.scaffold_195.1385 | *SYNJ2BP* | Synaptojanin-2-binding protein |
| evm.TU.scaffold_195.2 | *AGAP1* | Arf-GAP with GTPase, ANK repeat and PH domain-containing protein 1 |
| evm.TU.scaffold_197.220_evm.TU.scaffold_197.221 | *AFF3* | AF4/FMR2 family member 3 |
| evm.TU.scaffold_197.489 | *DYRK1A* | Dual specificity tyrosine-phosphorylation-regulated kinase 1A |
| evm.TU.scaffold_197.602 | *ROBO2* | Roundabout homolog 2 |
| evm.TU.scaffold_201.517 | *PNPLA7* | Patatin-like phospholipase domain-containing protein 7 |
| evm.TU.scaffold_209.17 | *IREB2* | Iron-responsive element-binding protein 2 |
| evm.TU.scaffold_209.288 | *CHIC2* | Cysteine-rich hydrophobic domain-containing protein 2 |
| evm.TU.scaffold_209.885 | *CLEC4M* | C-type lectin domain family 4 member M |
| evm.TU.scaffold_209.886 | *CLEC4C* | C-type lectin domain family 4 member C |
| evm.TU.scaffold_212.11 | *COL5A3* | Collagen alpha-3(V) chain |
| evm.TU.scaffold_212.3 | *CHCHD5* | Coiled-coil-helix-coiled-coil-helix domain-containing protein 5 |
| evm.TU.scaffold_212.4 | *LMX1B* | LIM homeobox transcription factor 1-beta |
| evm.TU.scaffold_212.6 | *NA* | Hepatic lectin |
| evm.TU.scaffold_212.7 | *NA* | Hepatic lectin |
| evm.TU.scaffold_251.48_evm.TU.scaffold_251.47 | *ELF1* | ETS-related transcription factor Elf-1 |
| evm.TU.scaffold_270.500 | *ARMC3* | Armadillo repeat-containing protein 3 |
| evm.TU.scaffold_271.8 | *C7ORF57* | Uncharacterized protein C7orf57 |
| evm.TU.scaffold_271.9 | *SUN3* | SUN domain-containing protein 3 |
| evm.TU.scaffold_291.94 | *DDX58* | Probable ATP-dependent RNA helicase DDX58 |
| evm.TU.scaffold_291.96 | *ACO1* | Cytoplasmic aconitate hydratase |
| evm.TU.scaffold_49.12 | *MRPL15* | 39S ribosomal protein L15, mitochondrial |
| evm.TU.scaffold_49.2 | *TMEM68* | Transmembrane protein 68 |
| evm.TU.scaffold_52.1139 | *PWWP2A* | PWWP domain-containing protein 2A |
| evm.TU.scaffold_52.1140 | *TTC1* | Tetratricopeptide repeat protein 1 |
| evm.TU.scaffold_52.1213 | *EPHX3* | Epoxide hydrolase 3 |
| evm.TU.scaffold_52.1214 | *BRD4* | Bromodomain-containing protein 4 |
| evm.TU.scaffold_52.1215 | *AKAP8* | A-kinase anchor protein 8 |
| evm.TU.scaffold_52.1261 | *FBXW10* | F-box/WD repeat-containing protein 10 |
| evm.TU.scaffold_52.1594 | *RARG* | Retinoic acid receptor gamma |
| evm.TU.scaffold_52.1596 | *ITGB7* | Integrin beta-7 |
| evm.TU.scaffold_52.1691 | *BPTF* | Nucleosome-remodeling factor subunit BPTF |
| evm.TU.scaffold_52.1725 | *ABCA5* | ATP-binding cassette sub-family A member 5 |
| evm.TU.scaffold_52.1729 | *ABCA8B* | ATP-binding cassette sub-family A member 8-B |
| evm.TU.scaffold_52.1730 | *NA* | NA |
| evm.TU.scaffold_52.1994 | *ARHGEF12* | Rho guanine nucleotide exchange factor 12 |
| evm.TU.scaffold_52.392 | *FOXP1* | Forkhead box protein P1 |
| evm.TU.scaffold_52.481 | *PLXNA1* | Plexin-A1 |
| evm.TU.scaffold_52.532 | *FAM208A* | Protein FAM208A |
| evm.TU.scaffold_52.533 | *CCDC66* | Coiled-coil domain-containing protein 66 |
| evm.TU.scaffold_52.602 | *ATP2B2* | Plasma membrane calcium-transporting ATPase 2 |
| evm.TU.scaffold_80.289 | *PRDM16* | PR domain zinc finger protein 16 |
| evm.TU.scaffold_84.1851 | *SYT14* | Synaptotagmin-14 |
| evm.TU.scaffold_84.2158 | *PARK2* | E3 ubiquitin-protein ligase parkin |
| evm.TU.scaffold_93.367 | *HOXB8* | Homeobox protein Hox-B8 |
| evm.TU.scaffold_93.669 | *VMN2R26* | Vomeronasal type-2 receptor 26 |
| evm.TU.scaffold_99.395 | *C16ORF59* | NA |
| evm.TU.scaffold_99.544 | *MCTP2* | Multiple C2 and transmembrane domain-containing protein 2 |
| evm.TU.scaffold_99.871 | *THSD4* | Thrombospondin type-1 domain-containing protein 4 |

**Table S14.** The top 1% genes under select sweep region in the Qinghai (QH) population. The genes marked in red were detected under strong positive selection using *F*_ST_, *θπ*, and XP-CLR.

| **Gene ID** | **Name** | **Description** |
| --- | --- | --- |
| evm.TU.scaffold_115.614 | *DDX20* | Probable ATP-dependent RNA helicase DDX20 |
| evm.TU.scaffold_129.293 | *DENND5B* | DENN domain-containing protein 5B |
| evm.TU.scaffold_129.294 | *METTL20* | Protein N-lysine methyltransferase METTL20 |
| evm.TU.scaffold_129.872 | *TMEM168* | Transmembrane protein 168 |
| evm.TU.scaffold_129.873 | *C7ORF60* | Probable methyltransferase BTM2 homolog |
| evm.TU.scaffold_157.45 | *PITPNM2* | Membrane-associated phosphatidylinositol transfer protein 2 |
| evm.TU.scaffold_157.47 | *NA* | NA |
| evm.TU.scaffold_157.48 | *OGFOD2* | 2-oxoglutarate and iron-dependent oxygenase domain-containing protein 2 |
| evm.TU.scaffold_157.64 | *P2RX4* | P2X purinoceptor 4 |
| evm.TU.scaffold_157.66 | *CAMKK2* | Calcium/calmodulin-dependent protein kinase kinase 2 |
| evm.TU.scaffold_168.1063 | *UBE2D2* | Ubiquitin-conjugating enzyme E2 D2 |
| evm.TU.scaffold_168.601 | *ESCO1* | N-acetyltransferase ESCO1 |
| evm.TU.scaffold_168.602 | *GREB1L* | GREB1-like protein |
| evm.TU.scaffold_195.1207 | *NELL1* | Protein kinase C-binding protein NELL1 |
| evm.TU.scaffold_195.1768 | *FAM181A* | Protein FAM181A |
| evm.TU.scaffold_195.1769 | *ASB2* | Ankyrin repeat and SOCS box protein 2 |
| evm.TU.scaffold_195.1849 | *MARK3* | MAP/microtubule affinity-regulating kinase 3 |
| evm.TU.scaffold_195.944 | *SYT12* | Synaptotagmin-12 |
| evm.TU.scaffold_197.310 | *ARHGAP6* | Rho GTPase-activating protein 6 |
| evm.TU.scaffold_197.603 | *ROBO1* | Roundabout homolog 1 |
| evm.TU.scaffold_197.874 | *MICU2* | Calcium uptake protein 2, mitochondrial |
| evm.TU.scaffold_209.533 | *LARP1B* | La-related protein 1B |
| evm.TU.scaffold_209.534 | *NA* | Uncharacterized protein C4orf29 homolog |
| evm.TU.scaffold_212.125 | *KRT75* | Keratin, type II cytoskeletal 75 |
| evm.TU.scaffold_259.2_evm.TU.scaffold_259.1 | *GRIK2* | Glutamate receptor ionotropic, kainate 2 |
| evm.TU.scaffold_270.375 | *PHF14* | PHD finger protein 14 |
| evm.TU.scaffold_291.13 | *DMRT2* | Doublesex- and mab-3-related transcription factor 2 |
| evm.TU.scaffold_291.148 | *SUSD1* | Sushi domain-containing protein 1 |
| evm.TU.scaffold_52.1293 | *DHRS7C* | Dehydrogenase/reductase SDR family member 7C |
| evm.TU.scaffold_52.1295 | *NARF* | Nuclear prelamin A recognition factor |
| evm.TU.scaffold_52.1728 | *ABCA5* | ATP-binding cassette sub-family A member 5 |
| evm.TU.scaffold_52.1729 | *ABCA8B* | ATP-binding cassette sub-family A member 8-B |
| evm.TU.scaffold_52.1800 | *NA* | NA |
| evm.TU.scaffold_52.1802 | *CCDC15* | Coiled-coil domain-containing protein 15 |
| evm.TU.scaffold_52.1982 | *MFRP* | Membrane frizzled-related protein |
| evm.TU.scaffold_52.1984 | *USP2* | Ubiquitin carboxyl-terminal hydrolase 2 |
| evm.TU.scaffold_52.821 | *SPINK1* | Pancreatic secretory trypsin inhibitor |
| evm.TU.scaffold_52.822 | *SPINK5* | Serine protease inhibitor Kazal-type 5 |
| evm.TU.scaffold_80.1102 | *CTPS1* | CTP synthase 1 |
| evm.TU.scaffold_80.1241 | *CMKLR1* | Chemokine-like receptor 1 |
| evm.TU.scaffold_84.1602 | *NCOA1* | Nuclear receptor coactivator 1 |
| evm.TU.scaffold_84.1616_evm.TU.scaffold_84.1617 | *MSRA* | Mitochondrial peptide methionine sulfoxide reductase |
| evm.TU.scaffold_84.1618 | *PRSS55* | Serine protease 55 |
| evm.TU.scaffold_84.1649 | *GAL10* | Gallinacin-10 |
| evm.TU.scaffold_84.1669_evm.TU.scaffold_84.1667 | *BCL11A* | B-cell lymphoma/leukemia 11A |
| evm.TU.scaffold_84.1707 | *FSHR* | Follicle-stimulating hormone receptor |
| evm.TU.scaffold_84.312 | *FAM13A* | Protein FAM13A |
| evm.TU.scaffold_84.313 | *SLC16A9* | Monocarboxylate transporter 9 |
| evm.TU.scaffold_84.315 | *CCDC6* | Coiled-coil domain-containing protein 6 |
| evm.TU.scaffold_84.420 | *ASCC1* | Activating signal cointegrator 1 complex subunit 1 |
| evm.TU.scaffold_84.444 | *PRKG1* | cGMP-dependent protein kinase 1 |
| evm.TU.scaffold_93.729 | *PRODH2* | Probable proline dehydrogenase 2 |
| evm.TU.scaffold_93.730 | *CABP5* | Calcium-binding protein 5 |
| evm.TU.scaffold_93.731 | *ALL2124* | Uncharacterized WD repeat-containing protein all2124 |
| evm.TU.scaffold_93.732 | *ADPRH* | [Protein ADP-ribosylarginine] hydrolase |

**Table S15.** Biological Process (BP) GO term enrichment results of selected genes in the NMG populations. Over-represented GO terms were defined as having at 1.5-fold enrichment and P ≤ 0.05 under Fisher's exact test.

| **GO_ID** | **GO_Term** | **P Value** | **Count** |
| --- | --- | --- | --- |
| GO:0016746 | transferase activity, transferring acyl groups | 3.60E-06 | 8 |
| GO:0004814 | arginine-tRNA ligase activity | 3.04E-04 | 2 |
| GO:0006420 | arginyl-tRNA aminoacylation | 3.04E-04 | 2 |
| GO:0008284 | positive regulation of cell proliferation | 9.09E-03 | 1 |
| GO:0030150 | protein import into mitochondrial matrix | 9.09E-03 | 1 |
| GO:0004689 | phosphorylase kinase activity | 1.11E-02 | 2 |
| GO:0005964 | phosphorylase kinase complex | 1.11E-02 | 2 |
| GO:0005978 | glycogen biosynthetic process | 1.36E-02 | 2 |
| GO:0031047 | gene silencing by RNA | 1.81E-02 | 1 |
| GO:0006621 | protein retention in ER lumen | 1.81E-02 | 1 |
| GO:0046923 | ER retention sequence binding | 1.81E-02 | 1 |
| GO:0033365 | protein localization to organelle | 1.86E-02 | 2 |
| GO:0005272 | sodium channel activity | 2.18E-02 | 2 |
| GO:0006629 | lipid metabolic process | 2.59E-02 | 5 |
| GO:0008093 | cytoskeletal adaptor activity | 2.70E-02 | 1 |
| GO:0017124 | SH3 domain binding | 2.70E-02 | 1 |
| GO:0046847 | filopodium assembly | 2.70E-02 | 1 |
| GO:0007596 | blood coagulation | 2.86E-02 | 3 |
| GO:0006801 | superoxide metabolic process | 3.15E-02 | 1 |
| GO:0046856 | phosphatidylinositol dephosphorylation | 4.03E-02 | 1 |
| GO:0005975 | carbohydrate metabolic process | 4.35E-02 | 6 |
| GO:0016311 | dephosphorylation | 4.71E-02 | 3 |

**Table S16.** KEGG enrichment results of selected genes in the NMG population. Over-represented GO terms were defined as having at 1.5-fold enrichment and P ≤ 0.05 under Fisher's exact test.

| **Map ID** | **Map Title** | **P Value** | **Count** |
| --- | --- | --- | --- |
| map04975 | Fat digestion and absorption | 1.23E-06 | 6 |
| map00561 | Glycerolipid metabolism | 6.89E-06 | 6 |
| map00100 | Steroid biosynthesis | 1.16E-04 | 4 |
| map00500 | Starch and sucrose metabolism | 5.22E-03 | 3 |
| map00380 | Tryptophan metabolism | 4.59E-02 | 2 |
| map04113 | Meiosis - yeast | 4.85E-02 | 2 |

**Table S17.** Biological Process (BP) GO term enrichment results of selected genes in the GS populations. Over-represented GO terms were defined as having at 1.5-fold enrichment and P ≤ 0.05 under Fisher's exact test.

| **GO_ID** | **GO_Term** | **P Value** | **Count** |
| --- | --- | --- | --- |
| GO:0009055 | electron carrier activity | 1.98E-03 | 5 |
| GO:0030246 | carbohydrate binding | 2.51E-03 | 4 |
| GO:0009428 | bacterial-type flagellum basal body, distal rod, P ring | 2.61E-03 | 3 |
| GO:0016887 | ATPase activity | 5.35E-03 | 7 |
| GO:0030288 | outer membrane-bounded periplasmic space | 9.62E-03 | 3 |
| GO:0005829 | cytosol | 9.62E-03 | 3 |
| GO:0001539 | cilium or flagellum-dependent cell motility | 1.63E-02 | 3 |
| GO:0004418 | hydroxymethylbilane synthase activity | 1.89E-02 | 1 |
| GO:0005388 | calcium-transporting ATPase activity | 1.89E-02 | 1 |
| GO:0007160 | cell-matrix adhesion | 1.99E-02 | 2 |
| GO:0000976 | transcription regulatory region sequence-specific DNA binding | 2.02E-02 | 3 |
| GO:1901363 | heterocyclic compound binding | 2.11E-02 | 31 |
| GO:0016881 | acid-amino acid ligase activity | 2.26E-02 | 2 |
| GO:0005550 | pheromone binding | 2.26E-02 | 1 |
| GO:0097159 | organic cyclic compound binding | 2.39E-02 | 31 |
| GO:0035176 | social behavior | 2.63E-02 | 1 |
| GO:0000062 | fatty-acyl-CoA binding | 3.00E-02 | 1 |
| GO:0004842 | ubiquitin-protein transferase activity | 3.56E-02 | 2 |
| GO:0046914 | transition metal ion binding | 4.04E-02 | 11 |
| GO:0016746 | transferase activity, transferring acyl groups | 4.08E-02 | 3 |
| GO:0007229 | integrin-mediated signaling pathway | 4.11E-02 | 1 |
| GO:0040011 | locomotion | 4.29E-02 | 5 |
| GO:0016597 | amino acid binding | 4.47E-02 | 1 |
| GO:0015934 | large ribosomal subunit | 4.47E-02 | 1 |
| GO:0017111 | nucleoside-triphosphatase activity | 4.49E-02 | 9 |
| GO:0004965 | G-protein coupled GABA receptor activity | 4.61E-02 | 2 |

**Table S18.** KEGG enrichment results of selected genes in the GS populations. Over-represented GO terms were defined as having at 1.5-fold enrichment and P ≤ 0.05 under Fisher's exact test.

| **Map ID** | **Map Title** | **P Value** | **Count** |
| --- | --- | --- | --- |
| map02010 | ABC transporters | 1.06E-07 | 6 |
| map00720 | Carbon fixation pathways in prokaryotes | 3.96E-03 | 2 |
| map01210 | 2-Oxocarboxylic acid metabolism | 3.96E-03 | 2 |
| map05162 | Measles | 4.22E-03 | 4 |
| map00020 | Citrate cycle (TCA cycle) | 1.23E-02 | 2 |
| map00630 | Glyoxylate and dicarboxylate metabolism | 1.52E-02 | 2 |
| map04975 | Fat digestion and absorption | 2.69E-02 | 2 |
| map04360 | Axon guidance | 3.48E-02 | 4 |
| map00625 | Chloroalkane and chloroalkene degradation | 3.88E-02 | 1 |
| map00561 | Glycerolipid metabolism | 4.59E-02 | 2 |

**Table S19.** Biological Process (BP) GO term enrichment results of selected genes in the QH populations. Over-represented GO terms were defined as having at 1.5-fold enrichment and P ≤ 0.05 under Fisher's exact test.

| **GO_ID** | **GO_Term** | **P Value** | **Count** |
| --- | --- | --- | --- |
| GO:0004867 | serine-type endopeptidase inhibitor activity | 2.06E-03 | 3 |
| GO:0008113 | peptide-methionine (S)-S-oxide reductase activity | 5.99E-03 | 1 |
| GO:0004657 | proline dehydrogenase activity | 5.99E-03 | 1 |
| GO:0006537 | glutamate biosynthetic process | 5.99E-03 | 1 |
| GO:0006562 | proline catabolic process | 5.99E-03 | 1 |
| GO:0003883 | CTP synthase activity | 8.98E-03 | 1 |
| GO:0051213 | dioxygenase activity | 9.14E-03 | 2 |
| GO:0035257 | nuclear hormone receptor binding | 1.20E-02 | 1 |
| GO:0016165 | linoleate 13S-lipoxygenase activity | 2.38E-02 | 1 |
| GO:0016874 | ligase activity | 2.77E-02 | 3 |
| GO:0019062 | virion attachment to host cell | 2.99E-02 | 2 |
| GO:0016525 | negative regulation of angiogenesis | 3.25E-02 | 1 |
| GO:0016879 | ligase activity, forming carbon-nitrogen bonds | 3.76E-02 | 2 |
| GO:0015097 | mercury ion transmembrane transporter activity | 4.41E-02 | 1 |
| GO:0015694 | mercury ion transport | 4.41E-02 | 1 |
| GO:0006275 | regulation of DNA replication | 4.41E-02 | 1 |
| GO:0009317 | acetyl-CoA carboxylase complex | 4.70E-02 | 1 |
| GO:0031418 | L-ascorbic acid binding | 4.70E-02 | 1 |

**Table S20.** KEGG enrichment results of selected genes in the QH populations. Over-represented GO terms were defined as having at 1.5-fold enrichment and P ≤ 0.05 under Fisher's exact test.

| **Map ID** | **Map Title** | **P Value** | **Count** |
| --- | --- | --- | --- |
| map02010 | ABC transporters | 9.34E-03 | 2 |
| map04080 | Neuroactive ligand-receptor interaction | 1.39E-02 | 4 |
| map05133 | Pertussis | 3.66E-02 | 2 |

**Table S21.** The primers for PCR.

| Species | Gene name | Forward primer (5'->3') | Reverse primer (5'->3') |
| --- | --- | --- | --- |
| Human | AKT1 | TGGGCAAGGGCACTTTCGG | AGGCGGTCGTGGGTCTGGAA |
| Human | TP53 | ACCACCATCCACTACAACTACAT | CACAAACACGCACCTCAAA |
| Human | CDK6 | TGCCCACTGAAACCATAAA | TACCACAGCGTGACGACCA |
| Human | CyclinD1 | TTCGGATGTTAGTGGCAGAG | ATTGATTCGCACGGTCAGG |
| Human | Fas | GCCAAGAAGGGAAGGAGTA | TGGTGTTGCTGGTGAGTGT |
| Human | Bcl2 | ATTGTGGCCTTCTTTGAGTTCG | CCTACCCAGCCTCCGTTATCC |
| Human | Casp3 | TTGATGCGTGATGTTTCTA | CAATGCCACAGTCCAGTTC |
| Human | Casp7 | GACCGAGCTTGATGATGGC | CTGGAACCGTGGAATAGGC |
| Human | APAF1 | ATGGCAGGCTGTGGGAAGT | TGCGGAGACGGTCTTTAGC |
| Human | ACTB-h | GAAGATCAAGATCATTGCTCCT | TACTCCTGCTTGCTGATCCA |
